# Source and Chemistry of Hydroxymethanesulfonate (HMS) in Fairbanks, Alaska

**DOI:** 10.1021/acs.est.2c00410

**Published:** 2022-05-11

**Authors:** James R. Campbell, Michael Battaglia, Kayane Dingilian, Meeta Cesler-Maloney, Jason M. St Clair, Thomas F. Hanisco, Ellis Robinson, Peter DeCarlo, William Simpson, Athanasios Nenes, Rodney J. Weber, Jingqiu Mao

**Affiliations:** † Geophysical Institute and Department of Chemistry and Biochemistry, University of Alaska Fairbanks, Fairbanks, Alaska 99775, United States; ‡ School of Earth and Atmospheric Sciences, Georgia Institute of Technology, Atlanta, Georgia 30332, United States; § Atmospheric Chemistry and Dynamics Laboratory, NASA Goddard Space Flight Center, Greenbelt, Maryland 20771, United States; ∥ Joint Center for Earth Systems Technology, University of Maryland Baltimore County, Baltimore, Maryland 21228, United States; ⊥ Department of Environmental Health and Engineering, Johns Hopkins University, Baltimore, Maryland 21218, United States; # Center for the Study of Air Quality and Climate Change, Institute of Chemical Engineering Sciences, Foundation for Research and Technology Hellas, Patras 26504, Greece; ∇ Laboratory of Atmospheric Processes and their Impacts, School of Architecture, Civil and Environmental Engineering, École Polytechnique Fédérale de Lausanne, Lausanne 1015, Switzerland

**Keywords:** hydroxymethanesulfonate, aerosol liquid water content, particulate pollution, online measurements, temperature inversions, particle-into-liquid sampler, ion chromatography

## Abstract

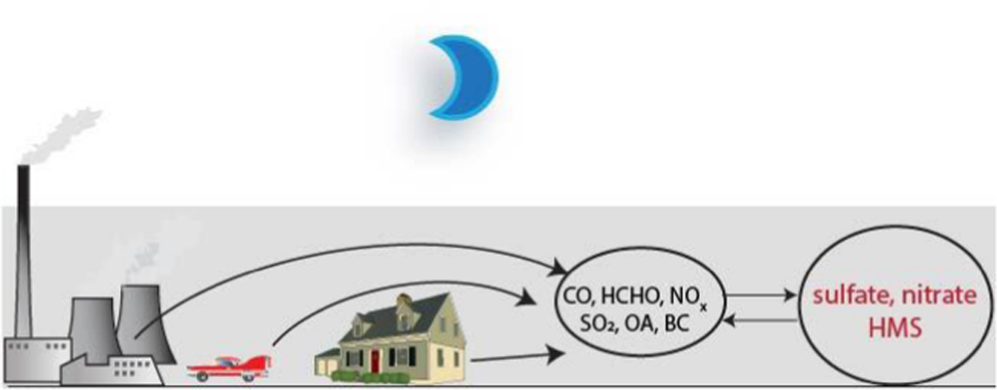

Fairbanks, Alaska, is a subarctic city with fine particle (PM_2.5_) concentrations that exceed air quality regulations in winter due to weak dispersion caused by strong atmospheric inversions, local emissions, and the unique chemistry occurring under the cold and dark conditions. Here, we report on observations from the winters of 2020 and 2021, motivated by our pilot study that showed exceptionally high concentrations of fine particle hydroxymethanesulfonate (HMS) or related sulfur(IV) species (e.g., sulfite and bisulfite). We deployed online particle-into-liquid sampler–ion chromatography (PILS-IC) in conjunction with a suite of instruments to determine HMS precursors (HCHO, SO_2_) and aerosol composition in general, with the goal to characterize the sources and sinks of HMS in wintertime Fairbanks. PM_2.5_ HMS comprised a significant fraction of PM_2.5_ sulfur (26–41%) and overall PM_2.5_ mass concentration of 2.8–6.8% during pollution episodes, substantially higher than what has been observed in other regions, likely due to the exceptionally low temperatures. HMS peaked in January, with lower concentrations in December and February, resulting from changes in precursors and meteorological conditions. Strong correlations with inorganic sulfate and organic mass during pollution events suggest that HMS is linked to processes responsible for poor air quality episodes. These findings demonstrate unique aspects of air pollution formation in cold and humid atmospheres.

## Abstract 2

Hydroxymethanesulfonate chemistry is poorly understood, despite making up a potentially large fraction of particulate pollution. These online measurements under cold and dark conditions reveal high concentrations and variability of hydroxymethanesulfonate.

## 1 Introduction

Fairbanks, Alaska (latitude 64.84°N), is a subarctic city that often has high mass concentrations of fine particulates (PM_2.5_, particles with an aerodynamic diameter of <2.5 μm) during the winter, resulting from limited dispersion due to strong and low altitude temperature inversions, local emissions, and possible gas-to-particle conversion processes under cold and dark conditions. Sulfate is estimated to account for roughly 15–25% of the PM_2.5_ mass, making it the second most abundant species, following organic compounds.^1−3^ Space heating is a major source of pollutants, and source apportionment studies have shown that woodsmoke contributes 40–70% to the wintertime PM_2.5_ mass concentration.^4^ Heating oil is thought to be the main source of sulfur,^4^ although it remains unclear how much particulate (hereon referred to as aerosol) sulfate is directly emitted (primary sulfate) over that formed in the atmosphere (secondary sulfur).

Recent studies suggest that hydroxymethanesulfonate (HMS) might be an overlooked component of sulfur chemistry, especially under cold and dark conditions.^5^ HMS is an adduct of dissolved sulfur dioxide (SO_2_) and formaldehyde (HCHO) and may undergo further chemistry to produce particulate sulfate. It is a strong acid, with a p*K*_a_ of <0 at 273 K.^6^ HMS has been found in fog and cloud water^7−10^ and aerosol particles^11−13^ and is thought to form mainly in the aqueous phase in the presence of SO_2_ and HCHO^14^
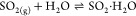
R1
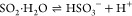
R2
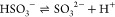
R3
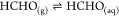
R4
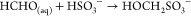
R5
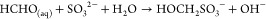
R6

R2 through R6 all occur in the aqueous phase. As shown in Table S1, the forward reaction rate constant for R6 is about 5 orders of magnitude faster than the one for R5, meaning that HMS formation occurs primarily through the reaction of HCHO and sulfite (SO_3_^2–^). Sulfite concentrations are highly sensitive to the pH of the aqueous solution, making HMS formation by this mechanism highly sensitive to pH as well.

Moderate pH (4–6) and high liquid water content in cloud/fog water provide favorable conditions for HMS formation.^8,9,13,15,16^ Like inorganic sulfate formed in cloud droplets, the low volatility of HMS means that it remains in the aerosol particle after the cloud droplets evaporate. As aerosol water concentration is several orders of magnitude lower than that of cloud/fog water, HMS production in the aerosol phase has generally been assumed to be negligible,^5^ but the extent to which this is true remains unclear. Song et al.^16^ proposed a mechanism similar to the one mentioned above for reactions in the aerosol phase. The decomposition of HMS back to its precursors is considered to be slow, taking place on the order of days.^6^

Quantification of HMS in ambient aerosols remains challenging. Despite HMS being previously measured by single-particle mass spectrometry (MS), ion chromatography (IC), and nuclear magnetic resonance (NMR),^10,17^ several studies have identified challenges with MS and IC methods. This may have led to substantial undermeasurement of PM_2.5_ HMS since the methods often combine HMS with sulfate, thus including it as part of the reported sulfate concentration. For example, most HMS peaks in MS are shared with either sulfate or common organic species, with little to no unique fragments.^11,18^ For IC, the detection of HMS is largely dependent on the pH of the eluent (mobile phase). For the commonly used Dionex anion IC with KOH as the eluent, the pH in the IC column can be 12 or higher, largely converting HMS to sulfate within the IC column, leading to a bias in the quantification of HMS.^18^ Depending on the column and eluents used, the IC also may not be able to fully separate the HMS and sulfate peaks. Both cases can lead to an underestimation of HMS and an overestimation of sulfate.^11,18^ Additionally, some fraction of HMS may be converted to sulfate in the sample preparation prior to injection into the IC for analysis. Most methods do not separate HMS from other S(IV) species, such as sulfite and bisulfite (SO_3_^2–^ and HSO_3_^–^), yet the peak is often quantified with pure HMS standards and referred to as HMS. Some groups have used dilute H_2_O_2_ in filter extractions to quantify HMS and total S(IV) separately and found that sulfite/bisulfite contribution to total S(IV) was negligible.^19−21^ Ma et al.^11^ used a similar method with dilute nitric acid and also found that sulfite/bisulfite contribution was negligible.

HMS measurements have mainly been made using offline methods utilizing aerosol filter samples. Most measurements in the US and Europe show low levels of HMS, often less than 0.1 μg/m^3^.^20,22,23^ Moch et al.^12^ found evidence of HMS in 139 of 158 IMPROVE network sites (one in Canada and South Korea and the rest in the US). They found average concentrations of 2.5 μg/m^3^ in Shijiazhuang, China, 0.50 μg/m^3^ in Singapore, and 0.05 μg/m^3^ in Po Valley, Italy. The analysis for IMPROVE, Shijiazhuang, and Singapore used IC with carbonate/bicarbonate eluents, while HMS in Po Valley was detected via NMR. The highest concentrations of HMS have been reported in wintertime China when PM_2.5_ mass concentrations were very high; Ma et al.^11^ found averages of 4 μg/m^3^ in 2015 and 7 μg/m^3^ in 2016 during severe winter haze in Beijing, peaking at 18.5 μg/m^3^. They reported that HMS accounted for an average of 1.5 and 2.7% of the PM_2.5_ mass concentration in 2015 and 2016, respectively, and the average HMS/sulfate molar ratios were 0.06 and 0.15 in 2015 and 2016, respectively. This is consistent with other studies, which range from 0.2 to 11%.^6^

Here, we describe the findings from deploying a particle-into-liquid sampler–ion chromatography (PILS-IC) system for the first in situ measurements of HMS in Fairbanks. We combine these measurements with an aerosol chemical speciation monitor (ACSM) for a broad chemical speciation of PM_2.5_ and measurements of formaldehyde (HCHO) and sulfur dioxide (SO_2_), to examine HMS formation during Fairbanks winters.

## 2 Measurements and Thermodynamic Analysis

### 2.1 Measurement Sites

We conducted our PILS-IC and HCHO measurements in a trailer near the UAF CTC (University of Alaska Fairbanks Community and Technical College, 64.84064°N, 147.72677°W, elevation 136 m above sea level) building in downtown Fairbanks for two winters: from January to March 2020 (winter 2020) and December 2020 to February 2021 (winter 2021). We also used hourly routine measurements of PM_2.5_ mass, SO_2_, and temperature made at the Alaska Department of Environmental Conservation’s (ADEC) NCore site, located at about 500 m from the CTC trailer (Figure S1). Next to the NCore site, another trailer housed the ACSM. As relative humidity (RH) measurements were not available at the NCore site, we used the RH measured at the Fairbanks International Airport for both winters (a map of locations is presented in Figure S1).

### 2.2 Instrument Methods

S(IV), which is quantified and referred to here as HMS, and sulfate were measured online using a PILS coupled with a Metrohm 761 IC (Metrohm USA, Riverside, FL). The detection limit for most anions (including sulfate) is roughly 10 ng/m^3^. With our setup, HMS has a retention time of about 15.5 min and a higher detection limit of around 150 ng/m^3^. The retention time of sulfate is about 1 min after HMS, causing some peak overlap that can affect the quantification of mainly HMS (Figure S2). To minimize this, the lowest point between the overlapping species was used to delineate the peaks, and the baseline for peak integration was drawn from this point to each peak’s unaffected baseline. This results in an error of less than approximately 20%, depending on their relative concentrations. The PILS sampling inlet was about 4 m above the snow-covered ground level. Stainless-steel sample tubing was fitted with a PM_2.5_ cyclone followed by an activated charcoal denuder and a glass honeycomb sodium carbonate-coated denuder, all located outside the trailer at ambient temperature.

Briefly, sample air is brought into the PILS at a flow of nominally 16.7 L/min and is mixed with a water vapor jet near 100 °C; the liquid water flow rate for the steam generator ranged from 0.25 to 0.75 mL/min. Rapid adiabatic cooling of the steam flow causes the water vapor to become supersaturated and condense on the sampled aerosol particles, which causes aerosols to grow into droplets large enough to be collected using an impactor. A liquid flow (transport flow) continuously washes the perimeter of the impaction plate where the collected droplets accumulate, and the combined flows are directed to the IC, allowing for the analysis of particles collected in the liquid sample. The transport flow contains 100 ppb LiBr as an internal standard. By comparing the concentration of Br^–^ in the transport flow to the overall resulting liquid sample exiting the PILS, the contribution of other sources of liquid (drops and steam condensate on the impaction plate) is determined and referred to as the dilution factor. The ambient concentration of the aerosol is calculated from the overall liquid flow, dilution factor, and sample air-flow rate, as described by Orsini et al.^24^ Our Br^–^ dilution values, which are calculated as the Br^–^ concentration entering the impactor divided by the concentration exiting, typically ranged from 1.2 to 1.3. In wintertime Fairbanks, the exceptionally cold ambient air mixing with steam within the PILS leads to a higher water supersaturation^24^ and higher condensational particle growth (more water vapor uptake) than in previous studies where ambient temperatures were more moderate. To avoid overdilution of the internal standard, we reduce the steam flow to maintain a dilution factor below 1.4.

The chromatographic method used involved isocratic elution on a Metrosep A Supp-5, 150/4.0 anion column at an eluent flow rate of 0.7 mL/min and pressure of 8.5 MPa. The IC anion eluent was 1.0 mM NaHCO_3_ and 3.2 mM Na_2_CO_3_, with a pH of nominally 10.5. This separates chloride, acetate/formate (coelute), bromide, nitrite, nitrate, and S(IV) that includes HMS, sulfate, and oxalate in about 23 min.

Calibrations for sulfate and most other anions were performed using commercially available liquid standards (IV-STOCK-59 from Inorganic Ventures). The HMS standard was prepared gravimetrically from solid formaldehyde–sodium bisulfite adduct 95% from Sigma-Aldrich, which had been dried within a desiccator. Calibrations for sulfate and HMS were done separately (i.e., HMS and sulfate were not combined into one standard). Full calibrations were done at concentrations of 10, 50, 100, 500, and 1000 ppb for both. A linear calibration plot was fitted to the peak area response of each species, which was then used to determine unknown concentrations of the species in samples. IC conductivity detection leads to an HMS sensitivity of about 4–5 times less than sulfate by mass. Our calibrations show that about 2–5% HMS mass is typically converted to sulfate during analysis (Figure S3); the data reported here have not been corrected to account for this. Furthermore, as noted above, the HMS reported here is defined as the sum of any species with the same retention time as that of HMS, including inorganic sulfite (SO_3_^2–^) and bisulfite (HSO_3_^–^). The reactions listed above (R1 through R6) show that SO_3_^2–^ and HSO_3_^–^ are HMS precursors, and estimates based on thermodynamic analysis suggest that their concentrations should be low for aerosol pH less than 6 (Figure S4). However, sulfite metal complexes are also known to exist in certain emissions.^25^ More work is needed to determine the speciation among these S(IV) components. In this study, we refer to the ion chromatographic S(IV) peak as HMS, recognizing that it may also include some of the HMS sulfur precursors.

Periodic calibrations were also done throughout the study to assess the measurement precision. A high-efficiency particulate air (HEPA) filter periodically placed on the inlet provided sample blanks (each blank with typically an average of 3–4 IC chromatograms). These were done approximately every 2 weeks and used to blank-correct all data and determine measurement uncertainty. Midway through our 2021 sampling period, we installed a new column. Full calibrations and periodic running of some standards were performed for each column throughout all ambient sampling periods.

### 2.3 Ancillary Measurements

Measurements of other species pertinent to this study were conducted at the ADEC NCore site (Figure S1). This includes temperature, SO_2_ (Thermo Scientific 43i-TLE), ozone (Teledyne API 400E), and PM_2.5_ mass concentration (Met-One BAM 1020X) measured at 4.5 m height. These data are averaged hourly and are available at the EPA database (https://aqs.epa.gov). As shown in Figure S1, the ADEC NCore site is about 500 m away from the CTC trailer. A previous study found that 24 h average aerosol filters of PM_2.5_ mass concentration and sulfate/PM_2.5_ mass ratio are not significantly different between the two sites.^4^ Previously, the ADEC monitoring site was the State Office Building, which was across the street from the CTC. When they moved from the State Office Building to the NCore, the ADEC found measurements on 24 h average PM_2.5_ mass concentrations and hourly CO concentrations between the two sites to be nearly identical (https://dec.alaska.gov/media/1710/2013-14-air-monitoring-network-plan.pdf) and concluded that the NCore site is representative of downtown Fairbanks air pollution. We merge the measurements from these two sites for data analysis here. We also use the aerosol speciation data collected at the NCore site, which is available every 3rd day.

We use RH data recorded at the Fairbanks International Airport using NOAA (https://erddap.aoos.org/erddap/tabledap/gov_noaa_nws_pafa.html). The airport is about 3 miles from NCore and CTC sites (Figure S1). Ambient temperature between the airport and NCore site are in close agreement, and so the RH at the airport is expected to be representative of that at the NCore site (Figure S5). The dynamic range of RH in wintertime Fairbanks is generally small since the ambient water vapor is often at the saturation water vapor pressure with respect to ice,^26^ not with respect to liquid water.

In 2020, gas-phase HCHO was measured at the CTC trailer using the NASA Goddard compact formaldehyde fluorescence experiment (COFFEE) instrument via a nonresonant laser-induced fluorescence (NR-LIF) technique.^27,28^ The NR-LIF technique uses a 355 nm laser to excite HCHO molecules and detects the resulting fluorescence in the visible light region (∼420–500 nm), providing a highly sensitive (1σ of ∼200 pptv at 0 ppbv HCHO) measurement at 1 s time resolution. The data have been averaged to 1 min for this analysis.

The ACSM, with a PM_2.5_ aerodynamic lens^29^ and capture vaporizer,^30^ provided measurements of the composition of PM_2.5_ (instead of PM_1_ in earlier instruments).^31^ The ACSM data utilized here are ammonium and organic aerosol (OA) and it’s operationally defined sulfate (sum of all sulfur-containing species), which we refer to as ACSM-sulfate. A PM_2.5_ cut cyclone (URG) at ambient temperature was attached to the inlet, and the sample RH was maintained below 30% using a Nafion dryer located inside the sampling building just upstream of the ACSM. As standard ionization efficiency calibrations were not possible during deployment, the ACSM concentration data were divided by the slope of the regression between ACSM-measured sulfate and the sulfate measured by 24 h average EPA filter measurements. The sum of all ACSM-measured PM_2.5_ species was then compared to the total PM_2.5_ mass from filters as a secondary check.

### 2.4 Aerosol pH and ALWC

We use ISORROPIA-Lite,^32^ a new version of thermodynamic equilibrium model ISORROPIA (http://isorropia.epfl.ch),^33^ to compute the aerosol liquid water content (ALWC) and pH of bulk PM_2.5_. ISORROPIA-Lite is identical to the solution process of ISORROPIA II version 2.3 but assumes metastable aerosol and allows the water uptake from internally mixed hygroscopic organics to affect the semivolatile partitioning of inorganic species considered by ISORROPIA II. The water uptake of the organics is parameterized using the hygroscopicity parameter (κ) and κ-Kohler theory^34^ and is described in detail by Kakavas et al.^32^

Model input is based on PILS-measured nitrate, sulfate, chloride, and ACSM-measured ammonium and organic aerosols. We ran ISORROPIA-Lite in the forward mode with the assumption of particles in a metastable state. Since the hygroscopicity of the OA measured by ACSM was unknown, we used a range of κ values, κ_OA_ = 0.10 (typical of less-oxidized organic aerosol),^35^ 0.15 (typical of continental and biogenic OA),^36^ and 0.20 (typical of highly oxidized isoprene and biomass burning aerosol),^35−37^ and assumed an organic aerosol density of 1.4 g/cm^3^, corresponding to oxidized aerosol. HMS is a highly hygroscopic organic species but not detected by the ACSM, so it is explicitly added to the organic mass with an assumed κ_HMS_ = 0.60 (the same κ value as that of ammonium sulfate). As a result, the overall κ for organics, κ_org_, is computed by the relative fraction of organic aerosols measured by ACSM and HMS measured by PILS-IC. For a starting κ_OA_ of 0.15, this leads to average κ_org_ values of 0.158 and 0.153 in 2020 and 2021, respectively.

As gas-phase NH_3_ measurements were not available, we did three sensitivity runs with total NH*_x_* (= NH_3_ + NH_4_^+^) as factors of one, two, and three times measured ammonium, corresponding to a particle to particle + gas partitioning fraction of ammonium (ε) of 1, 0.5, and 0.33, respectively. We also did not consider the gas-phase contributions to total nitrate (nitric acid) and chloride (hydrochloric acid) since at moderate particle pHs (3–5) for the expected ALWC and temperatures of Fairbanks winter, partitioning of these semivolatiles is almost exclusively in the particle phase, so the particle concentrations are an approximation of the total (gas + particle) levels.^38,39^

Nonvolatile cations (NVCs) such as sodium, calcium, and magnesium are also not considered in the analysis. If considered in the analysis, NVCs would elevate the pH relative to our predicted values (our values are lower estimates of pH), but the effect is likely minor since the concentrations are low (Table S2)—although it would still affect the partitioning of NH_3_ to the aerosol phase by a nontrivial amount.^40^ The largest uncertainty in the calculated pH stems from the lack of ammonia gas-phase data, given that for many of the aerosol acidity levels predicted here (i.e., when pH is above 2 for the levels of ALWC encountered here), significant amounts of the total ammonia remain in the gas phase.^41,42^

Some assumptions are made in our analysis. First, the hygroscopic organic aerosols will remain internally mixed with the inorganic-rich phase of the aerosols. If there is phase separation, the ALWC will still be present to interact with the semivolatile partitioning of the inorganics, but the pH of the inorganic-rich aerosol will change; however, it likely will not be affected much more than what is expected for the κ = 0 sensitivity calculation (i.e., no organic water in the inorganic aerosol system). Second, we assume that the particles remain in a liquid state at all times and do not freeze, even at temperatures down to −35 °C. The latter is likely given that the aerosol is deliquesced, should not contain significant amounts of dust or other ice-nucleating material (that promotes freezing in the immersion mode), and requires lower temperatures than −35 °C to freeze homogeneously.^43^ Closure of ISORROPIA-Lite predictions of ALWC and inorganic partitioning fractions will be made in follow-up papers, when appropriate data are available.

## 3 Results and Discussion

### 3.1 Observations of HMS and Its Gas-Phase Precursors in Two Winters

**Figure 1:**
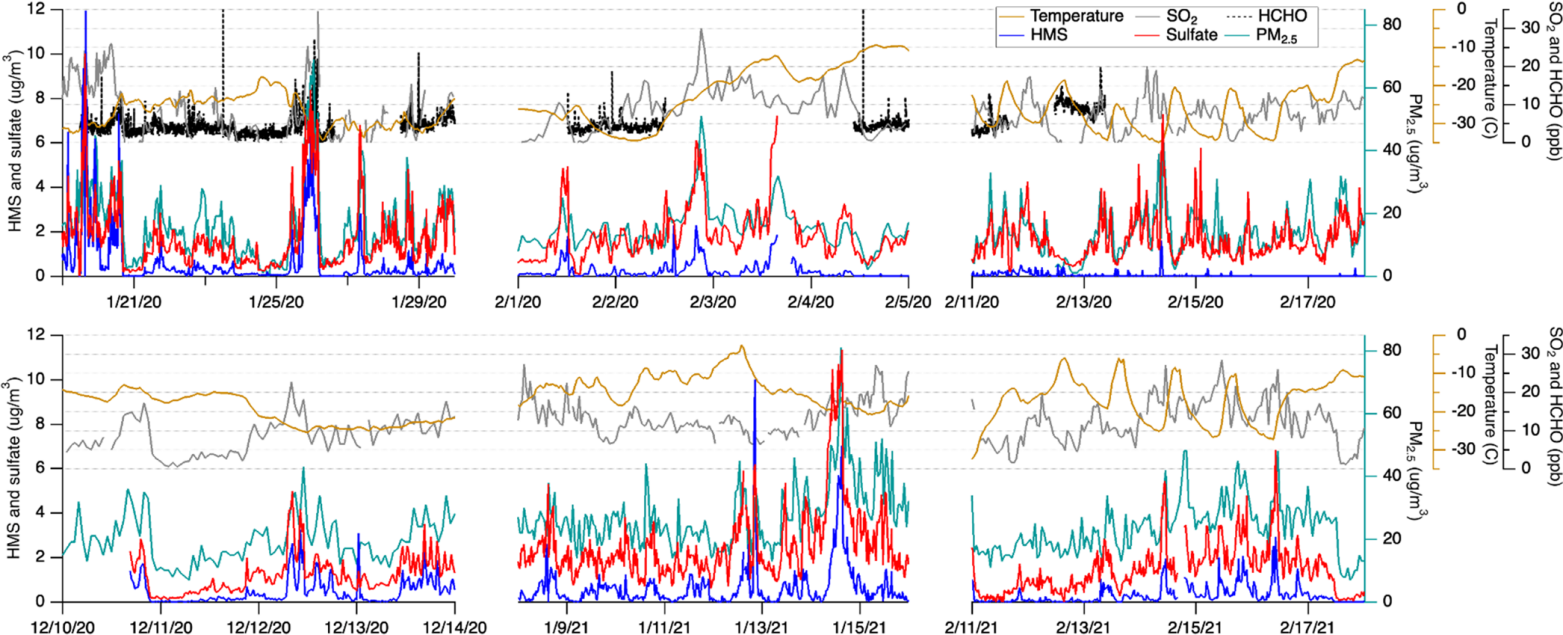
Time series of ambient temperature, gaseous HCHO and SO_2_, PM_2.5_ HMS and sulfate (by PILS), and PM_2.5_ mass in two winters, including (upper panel) January and February 2020 and (lower panel) December 2020 and January and February 2021.

Figure 1 shows the time series of measured PM_2.5_ HMS and sulfate mass, gaseous SO_2_ and HCHO, and ambient temperature during winter periods of 2020 and 2021. HMS was often above the measurement detection limit (0.15 μg/m^3^) throughout these times. High HMS concentrations were recorded even when ambient temperatures reached −35 °C in the January of 2020, which based on the current understanding of HMS formation mechanisms indicates the presence of particle liquid water. Quantifying HMS may help better understand the phase state of ambient aerosol particles in such a cold environment and assess the role of aqueous-phase chemistry in general.

HMS accounts for a major fraction of aerosol sulfur in the winter in Fairbanks when air quality levels are at their worst, suggesting that pollution episodes can lead to enhanced HMS production and at least some common processes between overall poor air quality and HMS production. Compared to severe haze events in Beijing, the only study where significant concentrations of HMS have been reported to date,^11^ the relative contributions of HMS to sulfate and PM_2.5_ mass are much higher in Fairbanks during pollution episodes. As shown in Table 1, the average HMS/sulfate molar ratios were 41% in 2020 and 26% in 2021 when hourly PM_2.5_ mass concentrations exceed 35 μg/m^3^ in Fairbanks, compared to 6–15% reported in Beijing during the local severe winter haze. The HMS/sulfate molar ratios in Fairbanks were 8.5% in 2020 and 11% in 2021 when hourly PM_2.5_ mass <35 μg/m^3^, similar to HMS/sulfate molar ratios under the most polluted conditions in Beijing. HMS in Fairbanks also accounts for a higher fraction of PM_2.5_ mass (3–7%) than that in Beijing (2–3%). HMS/OA ratios are comparable to those in previous studies, with 4.9% in Fairbanks in 2021 compared to 4.4–7.6% during severe winter haze in Beijing.^11^ However, during winter poor air quality events, overall pollutant concentrations in Beijing are higher than in Fairbanks, and this is true for HMS; average concentrations of HMS in Beijing during severe winter haze are in the range of 4–7 μg/m^3^ compared to 2.4 μg/m^3^ in 2020 and 1.3 μg/m^3^ in 2021 in Fairbanks when PM_2.5_ > 35 μg/m^3^.

**Table 1:** Summary of HMS, Sulfate, and PM_2.5_

	year	
	2020	2021
HMS (μg/m^3^)	0.29	0.34
HMS, PM_2.5_ > 35 (μg/m^3^)	2.36	1.27
HMS, PM_2.5_ < 35 (μg/m^3^)	0.20	0.25
sulfate (μg/m^3^)	1.66	1.56
HMS/sulfate (mol/mol)	0.11	0.12
HMS/sulfate, PM_2.5_ > 35 (mol/mol)	0.41	0.26
HMS/sulfate, PM_2.5_ < 35 (mol/mol)	0.09	0.11
PM_2.5_ (μg/m^3^)	10.2	21.8
SO_2_ (ppbv)	5.0	10.0

These *in situ* high-time resolution measurements reveal substantial temporal variability in HMS concentration. Most HMS spikes coincide with the peaks of SO_2_ and in some cases HCHO (where data are available), consistent with the importance of precursors (SO_2_, HCHO) on HMS formation. For example, during the period of Jan 25–27 of 2020, HMS concentrations reached 5 μg/m^3^, while SO_2_ concentrations peaked at 25 ppbv and those of HCHO peaked at 20 ppbv. During this HMS peak, HMS comprised 10% of the PM_2.5_ mass concentration and was at a similar concentration to that of ambient sulfate. However, HMS variability is not well-explained by SO_2_ and HCHO variability. During the period of Jan 14–16 of 2021, while SO_2_ steadily increased from 10 to 20 ppbv, HMS showed a sharp increase from 0 to 7 μg/m^3^ on Jan 14th and decreased shortly thereafter. In fact, this sharp increase in HMS appears to follow the trend of sulfate and ambient PM_2.5_ mass concentration more closely than SO_2_. We also see episodes when some SO_2_ spikes are not accompanied by an increase in HMS, such as the Feb 2nd of 2020. Generally, SO_2_ spikes only accompany an increase in HMS when PM_2.5_ mass concentration and/or sulfate also increases (Figure 1). One clear example of all chemical species increasing at once and temperature decreasing is between Jan 25 and 26 of 2020. As HCHO and SO_2_ concentrations in Fairbanks winter are comparable to the observations in Beijing (5–25 ppbv),^16^ the high HMS/sulfate ratio is likely due to factors beyond these precursors. Significant covariability in pollutants at this site can also be driven by the strength and height of the boundary layer.

Another notable feature of the data is the large month-to-month variability of HMS. We find that HMS reaches its highest concentrations in January, with lower concentrations in December and especially February. In 2020, the average HMS concentration for the entire sampling period decreased from 0.74 μg/m^3^ in January to 0.10 μg/m^3^ in February, an 87% decrease. A similar decrease of 63% was found in 2021 over these same months. This difference is likely in part due to the change under meteorological conditions. As shown in Figure 1, the ambient temperature shows little diurnal variation in December and January, while a much stronger diurnal variation was found in the second half of February of 2020 and 2021 as a result of increased solar heating and subsequently enhanced vertical mixing. Enhanced vertical mixing is also consistent with daily increases in ozone above the typical near-zero levels that occur in January and at night in February when ozone-rich air from aloft (due to the lack of NO*_x_* titration) mixes with surface air (Figure S6). With enhanced vertical mixing, a decrease of PM_2.5_ and SO_2_ is observed in the second half of February, along with lower levels of HMS. This observed month-to-month variability contradicts a previous model^12^ that predicted a summertime maximum of HMS in Alaska driven by cloud chemistry and little to no HMS in the winter.

### 3.2 HMS Correlation with Other Species

**Figure 2:**
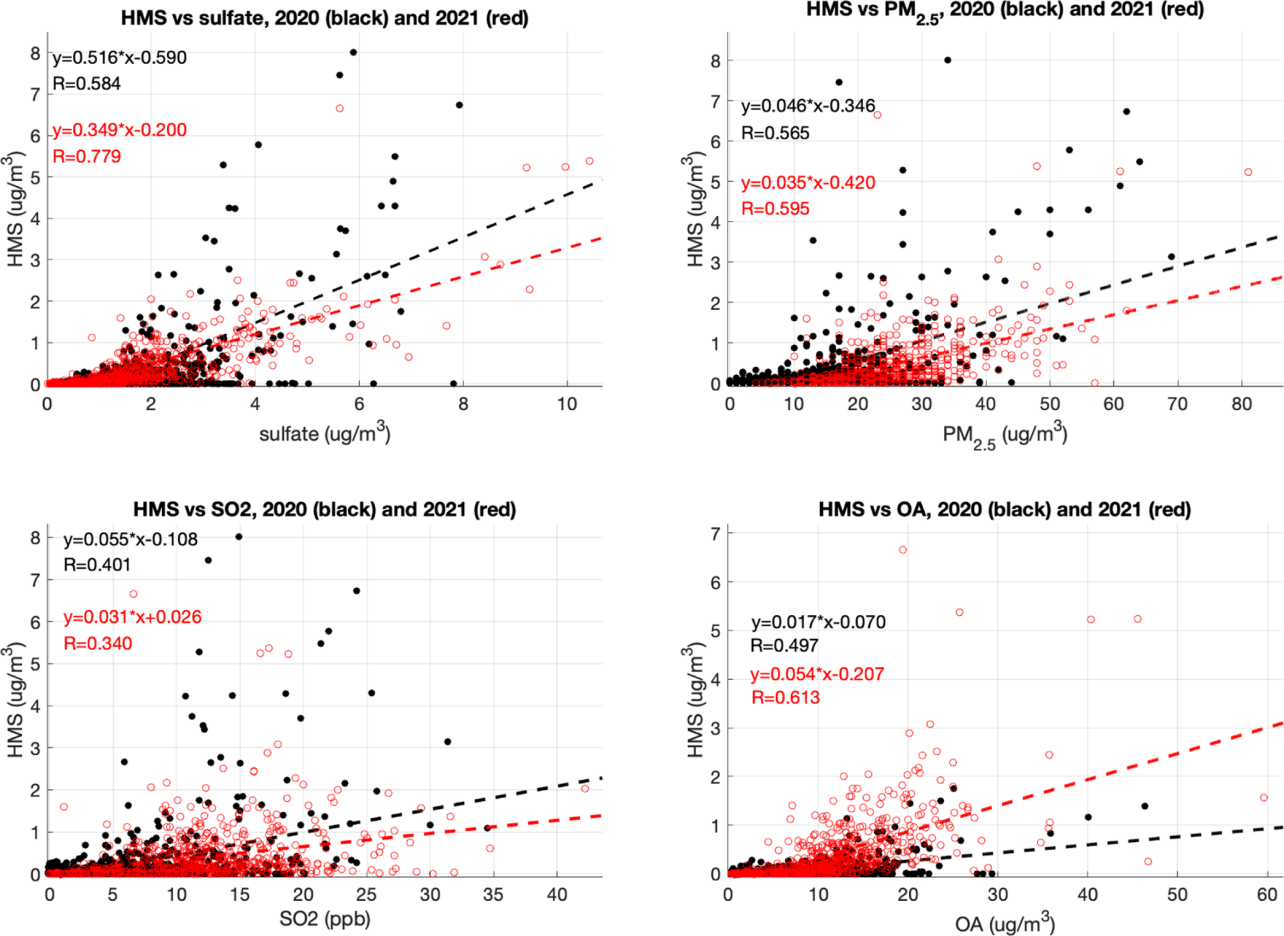
Correlations between the observed HMS and sulfate, PM_2.5_ mass, SO_2_, and PM_2.5_ OA for the winters of 2020 (black) and 2021 (red). OA data in 2020 were only available in February and March. York fit is applied here.^44^

Figure 2 shows the correlations between the observed HMS and sulfate, PM_2.5_ mass, SO_2_, and OA for the winters of 2020 and 2021. HMS correlates well with sulfate and PM_2.5_ mass concentration, with Pearson correlation coefficients (*R*) of 0.58 and 0.78 for sulfate and 0.57 and 0.60 for PM_2.5_ mass concentration in 2020 and 2021, respectively (Figure 2). The slopes of HMS vs sulfate and HMS vs PM_2.5_ mass concentration are similar between 2020 and 2021. The correlation between HMS and its possible precursor SO_2_ is weaker compared to that of sulfate and PM_2.5_ mass concentration, with correlation coefficients of 0.40 and 0.34 for SO_2_. Specifically, HMS concentrations tend to decrease when SO_2_ is over 20 ppbv, particularly for the year of 2021. In these cases, SO_2_ may not have undergone significant atmospheric processing and so was not depleted, and oxidation products like HMS would not have been formed. Thus, SO_2_ alone is not the dominant driver of HMS variability, and other significant precursors or rate-limiting processes in HMS formation play a significant role. Our good correlation between HMS and sulfate was also found in previous filter samples.^11,13,23^ HMS was also correlated with OA (*R* of 0.50 and 0.61), although the mass ratio of HMS to OA on average was small (2–5%).

The strong HMS–sulfate correlation may result from the availability of ALWC, as presumably both HMS and secondary sulfate are formed in aqueous phases. ISORROPIA-Lite calculations for κ as 0.15 predict that organic aerosols have a strong correlation with ALWC (Figure S7) and contribute 50–65% of ALWC. Sulfate contributes the second most at 21–33%, while nitrate and chloride contribute to 2–10 and 1–7%, respectively. Further sensitivity tests with κ as 0.1 and 0.2 give organic ALWC contributions of 40–58 and 56–72%, respectively, further suggesting a dominant role of organic aerosols in ALWC, largely due to the major contribution of organic aerosols to PM_2.5_ mass concentrations. As a contrast, the dominant role of organic aerosols in ALWC in Fairbanks is unique compared to the rural southeastern US, where OA contributed to 35% of the ALWC.^41^

Using the predicted ALWC, the instantaneous HMS production rate in aerosols can be estimated by
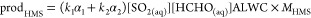
1where *k*_1_ and *k*_2_ are reaction rate constants for R5 and R6 respectively, [SO_2(aq)_] (the sum of SO_2_·H_2_O, bisulfite, and sulfite) and [HCHO_(aq)_] are aqueous-phase concentrations estimated from Henry’s law constants, α_1_ and α_2_ are the fraction of bisulfite and sulfite in [SO_2(aq)_], respectively, and *M*_HMS_ is the molar mass of HMS.^16^ Based on this, the production rate of HMS is in the range of 0–2 μg/m^3^/h (Figure S8), consistent with previous calculations,^13^ although our calculations may be associated with higher uncertainties at these lower temperatures. However, such slow production rate is at odds with the high temporal variability revealed by our measurements of HMS and its strong correlation with sulfate. Another possibility is that HMS may be formed in different locations and transported to the site, implying that the predicted time scales, which are based on conditions at the site, would not necessarily apply. For example, previous work shows that most HMS is predicted to form in regions of high ALWC (e.g., cloud or fogs); in Fairbanks, this may include exhaust plumes from vehicles and residential heating or plumes from the nearby coal-fired power plant cooling system (although emissions from elevated stacks are not expected to contribute much to ground-level pollution).^45^ Localized HMS production in these plumes would not be well-represented by the more average conditions observed at the sampling sites. However, primary sulfate or secondary sulfate formed within these plumes would remain in the particle phase along with any produced HMS as the water evaporates, maintaining covariability in sulfate and HMS at the site.

If HMS is indeed formed elsewhere, the strong HMS–sulfate correlation may also suggest simultaneous production of HMS and sulfate. In fact, a recent study suggests that sulfate formation can be rapid in the presence of NO_2_ and with aerosol pH in the range of 4–6.^46^ As NO_2_ is abundant in Fairbanks (>10 ppbv in pollution episodes), this mechanism provides an alternate explanation for simultaneous formation of both sulfate and HMS.^47^ Once sulfate is formed in the aerosol phase, this leads to a lower pH, which subsequently slows down both sulfate and HMS formation. Further modeling effort on HMS formation will be presented in a follow-up study.

### 3.3 Possible Drivers for HMS Formation

Factors other than precursors that may drive HMS variability are examined to provide insights into possible HMS formation routes.

#### 3.3.1 Ambient Temperature

**Figure 3:**
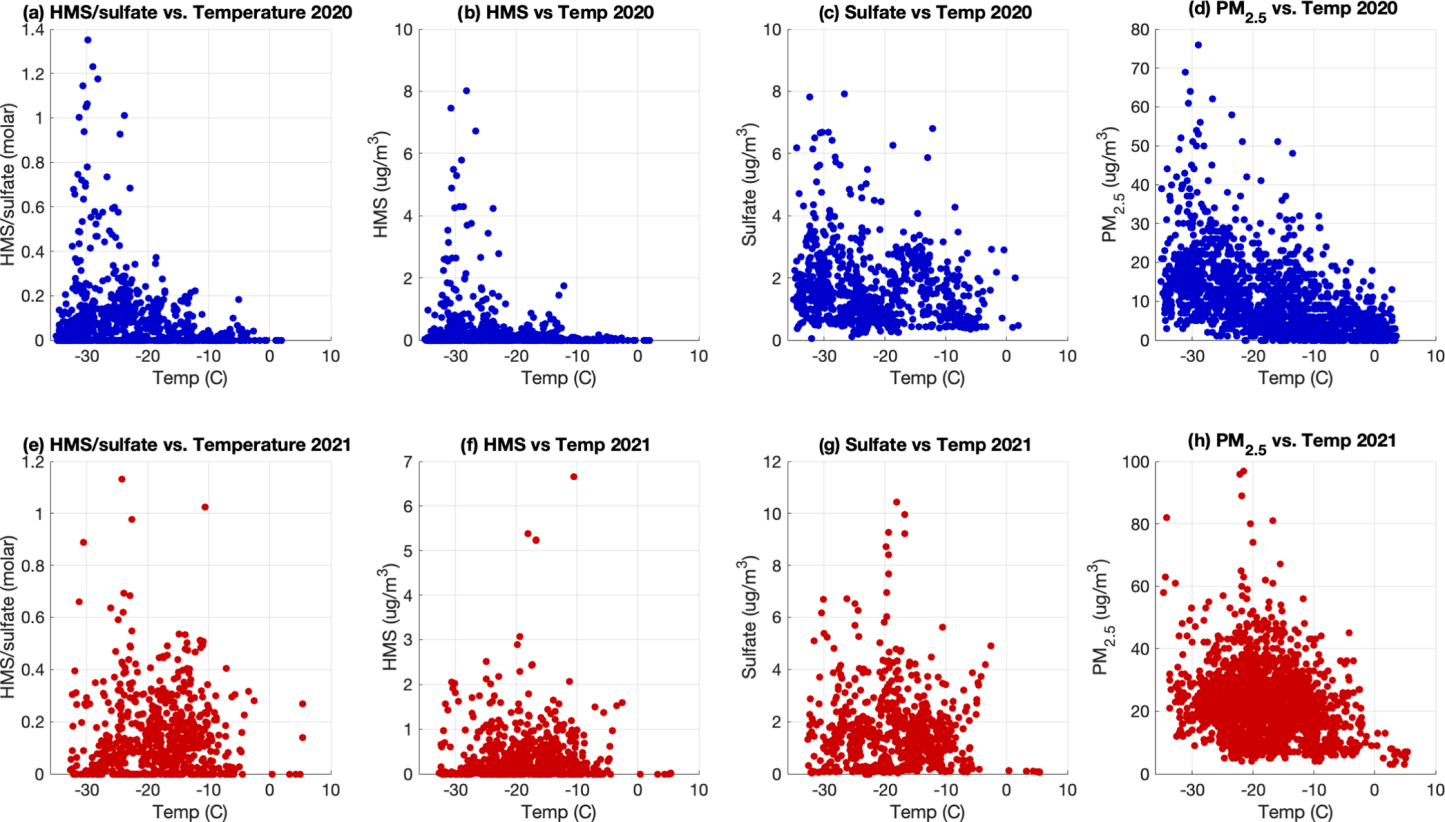
(a) HMS/sulfate molar ratio, (b) HMS, (c) sulfate, and (d) PM_2.5_ mass vs temperature in 2020. (e) HMS/sulfate molar ratio, (f) HMS, (g) sulfate, and (h) PM_2.5_ mass vs temperature in 2021.

Figure 3 shows significantly higher values of HMS in 2020 than in 2021. This could be due in part to more frequent extreme cold events during the 2020 sampling period (Figure S9). Lower temperature could significantly facilitate HMS production by enhancing the solubility of SO_2_ and HCHO. The effective Henry’s law constant for SO_2_ increases from 780 M/atm at 273 K to 1.6 × 10^4^ M/atm at 235 K (at pH = 4). The effective Henry’s law constant for HCHO increases from 1.8 × 10^5^ M/atm at 273 K to 7.8 × 10^7^ M/atm at 235 K. Thus, solubility of both SO_2_ and HCHO increases by 2–3 orders of magnitude, greatly enhancing the aqueous concentrations of S(IV) and HCHO. As a result, the aqueous-phase HMS production rate may increase by 5–6 orders of magnitude when the temperature decreases from 273 to 235 K. It is possible that this large increase in solubility with extremely low temperature may facilitate the HMS production in aerosol water, whereas this route was considered to be too slow in global modeling studies where urban-scale chemistry is poorly represented in global models.^6,12^ Also, as the ambient temperature in wintertime Fairbanks (often around −30 °C) is much lower than that of Beijing’s winter (around −5 to 0 °C, https://en.climate-data.org/asia/china/beijing/beijing-134/), this could also explain the observed difference in HMS/sulfate between the two urban areas.

However, extremely low temperature alone does not fully explain HMS variability. While Figure 3 shows a strong temperature dependence of HMS and the HMS/sulfate ratio in 2020, the temperature dependence of both appears to be much weaker in 2021. This is further illustrated with the time series in Figure 1. As an example, on the Feb 1st of 2020, the ambient temperature was nearly −35 °C, while HMS was less than 1 μg/m^3^. Meanwhile, between Jan 12 and 14 of 2021, the temperature ranged from around only −10 to −18 °C, but HMS reached its highest concentrations (10.0 μg/m^3^) of that winter. A similar phenomenon can be seen when comparing December 2020 to January 2021; although in December 2020, temperatures were on average lower, HMS concentrations were higher in January 2021. When temperature drop and PM_2.5_ mass concentration increase (often indicating a strong inversion event), we almost always see a spike in HMS. This suggests that the temperature dependence of HMS in 2020 is at least partly driven by the temperature dependence of sulfate and overall PM_2.5_ mass concentration. Another possibility is that at temperatures of around −35 °C, aerosols may become glassy aerosols,^48,49^ preventing gas-phase partitioning of SO_2_ and HCHO and thus significantly reducing the formation of HMS. However, an in-depth analysis would be needed when appropriate measurements are available.

#### 3.3.2 Relative Humidity

RH in Fairbanks winter (65–85%) has a much narrower dynamic range compared to that in studies done in Beijing (about 25–90%).^11^ As shown in Figure S10, the ambient RH is largely in the range of 70–80%, with a few days reaching 90%. This is because when the surface is covered by snow and ice, the water vapor pressure often reaches the saturation water vapor pressure with respect to ice^26^ instead of saturation water vapor pressure with respect to liquid water. Ma et al.^11^ likely had little to no snow coverage on the ground during sampling (https://www.timeanddate.com/weather/china/beijing/historic) and so had no RH contribution from snow or ice. We show in Figure S10 that HMS concentrations are not well-correlated with RH in 2020 and 2021. This is in contrast to previous findings in Beijing by Ma et al.,^11^ which show higher HMS concentrations with increasing RH. Within the narrow range of RH, we do not find RH as a main driver for HMS variability in Fairbanks winter. In this case, variability in ALWC may not be closely related to variability in RH but instead aerosol species (mainly sulfate and OA) that drive the particle water uptake.

#### 3.3.3 Ozone

Several studies have examined the role of ozone in HMS formation where ozone was used as an indicator of the ambient oxidant level.^6,11,13^ Consistent with these studies, we find a similar relationship between HMS and ozone, i.e., high HMS at low ozone levels (Figure S10). The anticorrelation here is driven largely by the physical location of ozone: it is at low concentrations below the inversion layer due to titration by NO*_x_*, whereas above the boundary layer, NO*_x_* is much lower and ozone is at background levels. We find that NO*_y_* correlates well with PM_2.5_ (NO*_x_* data were not available), thus correlating with HMS, which would drive the anticorrelation with ozone (Figure S11). In any case, this suggests that HMS is not likely formed from oxidation by O_3_ and the associated free radicals.

#### 3.3.4 ALWC

Sensitivity tests using ISORROPIA-Lite to predict ALWC suggest that under typical Fairbanks winter conditions (OA ∼ 12 μg/m^3^, sulfate ∼ 2 μg/m^3^, nitrate ∼ 0.7 μg/m^3^, ammonium ∼ 0.4 μg/m^3^, chloride ∼ 0.3 μg/m^3^, RH ∼ 70–80%), ALWC appears to be insensitive to ambient temperature at −5 °C and below (Figure S12d,h). The narrow range in RH (70–80%) suggests that ALWC would be strongly affected by variability in the concentration of hygroscopic aerosol species. ISORROPIA-Lite predicts that ALWC is highly correlated with the mass concentrations of OA even over a wide range of hygroscopicity (κ = 0.1–0.2), due to OA being the dominant PM_2.5_ component. Aerosol sulfate is the second largest component of PM_2.5_ and is highly hygroscopic. Thus, if HMS is formed in aerosol particle water and not in cloud or fog at another location, the HMS correlation with sulfate and OA (see Figure 2), to some extent, results from these species controlling the liquid water concentration. This implies that controlling the OA sources, such as wood burning, may also reduce HMS levels.

#### 3.3.5 Aerosol pH

ISORROPIA-Lite calculations show that aerosol pH largely ranges between 3 and 5 (which favors HMS formation), when ambient NH*_x_* is three times (ε = 0.33) that of ACSM-measured particle ammonium concentration (Figure S12). Aerosol pH can be significantly lower with lower amounts of NH*_x_* (ε of 0.5 and 1). Under these conditions, the periods of favorable HMS formation (pH 3–5) decrease. A plot of the measured HMS vs calculated pH is shown in Figure S13; with the exception of the case where NH*_x_* = 3 × NH_4_, there are still considerable amounts of data with pH less than 3 and with significant levels of HMS. This can be viewed as being inconsistent with what is known about HMS formation, given that its formation is not favored under strongly acidic conditions—even at low temperatures (Figure S8). Therefore, the NH*_x_* = NH_4_ and NH*_x_* = 2 × NH_4_ scenarios seem less probable than NH*_x_* = 3 × NH_4_, assuming that the conditions at the measurement site reflect the conditions under which HMS is formed. A more precise constraint on aerosol pH is required, which can be done by better constraining the gas-phase NH_3_ levels, either through a direct measurement or inference through auxiliary measurements.

### 3.4 Comparison between PILS-IC and ACSM

Two online measurements of sulfate were made during this study: the PILS-IC, which can distinguish HMS and sulfate, and the ACSM, which likely is not based on the analysis of an instrument that operates on a similar principle (aerosol mass spectrometer) in another study.^18^Figure S14 compares sulfate measurements by the ACSM and PILS in 2021. In the following paragraph, the sum of PILS-IC sulfate and HMS will be referred to as “PILS sulfur” for readability. The ACSM sulfate should be assumed to be the sum of all forms of PM_2.5_ sulfate, including HMS.^18^ In general, ACSM sulfate concentrations show a good correlation with PILS sulfate concentrations, but ACSM measurements are higher than PILS ones by roughly 10% (Pearson’s *R*^2^ = 0.65). However, ACSM sulfate compared to PILS sulfur shows a better correlation (Pearson’s *R*^2^ = 0.69) and brings the slope closer to 1. When PM_2.5_ > 25 μg/m^3^, the difference of ACSM sulfate and PILS sulfur is often around 5 μg/m^3^ but can be up to 12 μg/m^3^, with ACSM being higher. These results are consistent with the PILS separating sulfate and HMS and the ACSM sulfate being the sum of the two.

There may be additional causes for differences between the PILS and ACSM sulfur measurements. In some cases, the PILS sulfur is higher than ACSM sulfate when in the same PM_2.5_ range, but by only up to 3 μg/m^3^ or less. This may be an indicator that there is another sulfur species that becomes more prominent as PM_2.5_ increases—one that ACSM detects but PILS-IC does not. One possibility is the presence of other organosulfur species. This PILS-IC was not set up to measure organosulfur species, which are generally insoluble (no ions formed when collected into water) or not separated or retained by the IC columns utilized, meaning that they would not be detected by the PILS-IC. More detailed speciation of the organic species, including various organosulfates, would help understand the differences seen between these two instruments.

## 4 Atmospheric Implications

In summary, new online measurements of PM_2.5_ aerosol particle composition in Fairbanks, Alaska, during cold periods with instrumentation capable of separating sulfate and sulfur(IV) species such as hydroxymethanesulfonate (HMS) reveal exceptionally high levels of HMS relative to sulfate and PM_2.5_ mass. In some cases, HMS concentrations are comparable to those of sulfate, providing a large sulfur reservoir for ambient aerosols under cold and dark conditions. The presence of HMS also suggests the presence of liquid water in ambient aerosols, even at temperatures down to −35 °C. Analysis of the data suggests that anthropogenic sulfur emissions combined with high levels of organic aerosol (and hence overall PM_2.5_ mass) and consistent ambient relative humidity in the range of 70–80% provide significant amounts of ALWC. Combined with the extremely low wintertime temperatures in this subarctic urban environment that can enhance the aqueous uptake of precursor gases (SO_2_ and HCHO), the probable mildly acidic levels in the aerosol may be the cause for the high HMS concentrations. The ubiquity of these drivers suggests that HMS may be a common component of PM_2.5_ in other populated and polluted regions during extreme cold events. The possibly large role of organic species, largely from heating with wood and oil, is curious. Since the OA is a large mass fraction and likely mildly hygroscopic, it has a large effect on the overall concentration of particle water, which can enhance HMS production by increasing the reactor volume and raising the particle pH by dilution. OA from domestic wood burning is particularly important since it is a source of ammonia and cations, such as potassium, that also raises particle pH. This implies that strategies to mitigate OA, such as limiting domestic wood heating, may also reduce HMS and other species formed through aqueous processes. This may have contributed to our observations of high correlations between PM_2.5_ mass and many of the individual PM species in Fairbanks, although covariability due to meteorological effects can also be a major cause. Further studies on how HMS is formed in these unique environments and the prevailing acidity and ALWC levels would help further assess the insights obtained here and eventually shape effective emission policies on air quality. A better understanding of HMS chemistry may also shed light on multiphase chemistry, aerosol thermodynamics, and phase states in very cold regions in general, including the upper troposphere.

## Supporting Information Available

Location of field measurement sites; example IC chromatogram; HMS IC standard analysis; calculation of sulfite and bisulfite concentrations; comparison between NCore and airport temperatures; ozone diurnal cycle; ALWC vs OA and ALWC vs sulfate; calculated HMS production rate; temperature frequency for the sampling period; correlation between HMS and related measurements; comparison of PM_2.5_ and HMS with NO*_y_*; calculated pH vs sulfate and ALWC vs temperature; calculated pH vs HMS; comparison between PILS and ACSM measurements; Moch et al. (2018) GEOS-Chem implementation; and NVC concentrations (PDF)

## References

[ref1] Kotchenruther R. A. Source Apportionment of PM 2.5 at Multiple Northwest U.S. Sites: Assessing Regional Winter Wood Smoke Impacts from Residential Wood Combustion. Atmos. Environ. 2016, 142, 210–219. 10.1016/j.atmosenv.2016.07.048.

[ref2] Wang Y.; Hopke P. K. Is Alaska Truly the Great Escape from Air Pollution? – Long Term Source Apportionment of Fine Particulate Matter in Fairbanks, Alaska. Aerosol Air Qual. Res. 2014, 14, 1875–1882. 10.4209/aaqr.2014.03.0047.

[ref3] Ward T.; Trost B.; Conner J.; Flanagan J.; Jayanty R. K. M. Source Apportionment of PM2.5 in a Subarctic Airshed - Fairbanks, Alaska. Aerosol Air Qual. Res. 2012, 12, 536–543. 10.4209/aaqr.2011.11.0208.

[ref4] Nattinger K. C. Temporal and Spatial Trends of Fine Particulate Matter Composition in Fairbanks, Alaska; University of Alaska Fairbanks, 2016; p 144.

[ref5] Moch J. M.; Dovrou E.; Mickley L. J.; Keutsch F. N.; Cheng Y.; Jacob D. J.; Jiang J.; Li M.; Munger J. W.; Qiao X.; Zhang Q. Contribution of Hydroxymethane Sulfonate to Ambient Particulate Matter: A Potential Explanation for High Particulate Sulfur During Severe Winter Haze in Beijing. Geophys. Res. Lett. 2018, 45, 11969–11979. 10.1029/2018GL079309.

[ref6] Song S.; Ma T.; Zhang Y.; Shen L.; Liu P.; Li K.; Zhai S.; Zheng H.; Gao M.; Moch J. M.; Duan F.; He K.; McElroy M. B. Global Modeling of Heterogeneous Hydroxymethanesulfonate Chemistry. Atmos. Chem. Phys. 2021, 21, 457–481. 10.5194/acp-21-457-2021.

[ref7] Munger J. W.; Jacob D. J.; Waldman J. M.; Hoffmann M. R. Fogwater Chemistry in an Urban Atmosphere. J. Geophys. Res.: Oceans 1983, 88, 5109–5121. 10.1029/JC088iC09p05109.

[ref8] Munger J. W.; Jacob D. J.; Hoffmann M. R. The Occurrence of Bisulfite-Aldehyde Addition Products in Fog- and Cloudwater. J. Atmos. Chem. 1984, 1, 335–350. 10.1007/BF00053799.

[ref9] Munger J. W.; Tiller C.; Hoffmann M. R. Identification of Hydroxymethanesulfonate in Fog Water. Science 1986, 231, 247–249. 10.1126/science.231.4735.247. 17769644

[ref10] Whiteaker J. R.; Prather K. A. Hydroxymethanesulfonate as a Tracer for Fog Processing of Individual Aerosol Particles. Atmos. Environ. 2003, 37, 1033–1043. 10.1016/S1352-2310(02)01029-4.

[ref11] Ma T.; Furutani H.; Duan F.; Kimoto T.; Jiang J.; Zhang Q.; Xu X.; Wang Y.; Gao J.; Geng G.; Li M.; Song S.; Ma Y.; Che F.; Wang J.; Zhu L.; Huang T.; Toyoda M.; He K. Contribution of Hydroxymethanesulfonate (HMS) to Severe Winter Haze in the North China Plain. Atmos. Chem. Phys. 2020, 20, 5887–5897. 10.5194/acp-20-5887-2020.

[ref12] Moch J. M.; Dovrou E.; Mickley L. J.; Keutsch F. N.; Liu Z.; Wang Y.; Dombek T. L.; Kuwata M.; Budisulistiorini S. H.; Yang L.; Decesari S.; Paglione M.; Alexander B.; Shao J.; Munger J. W.; Jacob D. J. Global Importance of Hydroxymethanesulfonate in Ambient Particulate Matter: Implications for Air Quality. J. Geophys. Res.: Atmos. 2020, 125, e2020JD032706 10.1029/2020JD032706. PMC7685164 33282612

[ref13] Wei L.; Fu P.; Chen X.; An N.; Yue S.; Ren H.; Zhao W.; Xie Q.; Sun Y.; Zhu Q.-F.; Wang Z.; Feng Y.-Q. Quantitative Determination of Hydroxymethanesulfonate (HMS) Using Ion Chromatography and UHPLC-LTQ-Orbitrap Mass Spectrometry: A Missing Source of Sulfur during Haze Episodes in Beijing. Environ. Sci. Technol. Lett. 2020, 7, 701–707. 10.1021/acs.estlett.0c00528.

[ref14] Boyce S. D.; Hoffmann M. R. Kinetics and Mechanism of the Formation of Hydroxymethanesulfonic Acid at Low PH. J. Phys. Chem. A 1984, 88, 4740–4746. 10.1021/j150664a059.

[ref15] Olson T. M.; Hoffmann M. R. Hydroxyalkylsulfonate Formation: Its Role as a S(IV) Reservoir in Atmospheric Water Droplets. Atmos. Environ. 1989, 23, 985–997. 10.1016/0004-6981(89)90302-8.

[ref16] Song S.; Gao M.; Xu W.; Sun Y.; Worsnop D. R.; Jayne J. T.; Zhang Y.; Zhu L.; Li M.; Zhou Z.; Cheng C.; Lv Y.; Wang Y.; Peng W.; Xu X.; Lin N.; Wang Y.; Wang S.; Munger J. W.; Jacob D. J.; McElroy M. B. Possible Heterogeneous Chemistry of Hydroxymethanesulfonate (HMS) in Northern China Winter Haze. Atmos. Chem. Phys. 2019, 19, 1357–1371. 10.5194/acp-19-1357-2019.

[ref17] Neubauer K. R.; Sum S. T.; Johnston M. V.; Wexler A. S. Sulfur Speciation in Individual Aerosol Particles. J. Geophys. Res.: Atmos. 1996, 101, 18701–18707. 10.1029/96JD01555.

[ref18] Dovrou E.; Lim C. Y.; Canagaratna M. R.; Kroll J. H.; Worsnop D. R.; Keutsch F. N. Measurement Techniques for Identifying and Quantifying Hydroxymethanesulfonate (HMS) in an Aqueous Matrix and Particulate Matter Using Aerosol Mass Spectrometry and Ion Chromatography. Atmos. Meas. Tech. 2019, 12, 5303–5315. 10.5194/amt-12-5303-2019.

[ref19] Dabek-Zlotorzynska E.; Piechowski M.; Keppel-Jones K.; Aranda-Rodriguez R. Determination of Hydroxymethanesulfonic Acid in Environmental Samples by Capillary Electrophoresis. J. Sep. Sci. 2002, 25, 1123–1128. 10.1002/1615-9314(20021101)25:15/17<1123::AID-JSSC1123>3.0.CO;2-3.

[ref20] Dixon R. W.; Aasen H. Measurement of Hydroxymethanesulfonate in Atmospheric Aerosols. Atmos. Environ. 1999, 33, 2023–2029. 10.1016/S1352-2310(98)00416-6.

[ref21] Rao X.; Collett J. L. Jr. Behavior of S(IV) and Formaldehyde in a Chemically Heterogeneous Cloud. Environ. Sci. Technol. 1995, 29, 1023–1031. 10.1021/es00004a024. 22176411

[ref22] Liu J.; Gunsch M. J.; Moffett C. E.; Xu L.; El Asmar R.; Zhang Q.; Watson T. B.; Allen H. M.; Crounse J. D.; St Clair J.; Kim M.; Wennberg P. O.; Weber R. J.; Sheesley R. J.; Pratt K. A. Hydroxymethanesulfonate (HMS) Formation during Summertime Fog in an Arctic Oil Field. Environ. Sci. Technol. Lett. 2021, 8, 511–518. 10.1021/acs.estlett.1c00357.

[ref23] Scheinhardt S.; van Pinxteren D.; Müller K.; Spindler G.; Herrmann H. Hydroxymethanesulfonic Acid in Size-Segregated Aerosol Particles at Nine Sites in Germany. Atmos. Chem. Phys. 2014, 14, 4531–4538. 10.5194/acp-14-4531-2014.

[ref24] Orsini D. A.; Ma Y.; Sullivan A.; Sierau B.; Baumann K.; Weber R. J. Refinements to the Particle-into-Liquid Sampler (PILS) for Ground and Airborne Measurements of Water Soluble Aerosol Composition. Atmos. Environ. 2003, 37, 1243–1259. 10.1016/S1352-2310(02)01015-4.

[ref25] Eatough D. J.; Major T.; Ryder J.; Hill M.; Mangelson N. F.; Eatough N. L.; Hansen L. D.; Meisenheimer R. G.; Fischer J. W. The Formation and Stability of Sulfite Species in Aerosols. In Sulfur in the Atmosphere; Elsevier, 1978; pp 263–271.

[ref26] Andreas E. L.; Guest P. S.; Persson P. O. G.; Fairall C. W.; Horst T. W.; Moritz R. E.; Semmer S. R. Near-Surface Water Vapor over Polar Sea Ice Is Always near Ice Saturation. J. Geophys. Res.: Oceans 2002, 107, SHE 8-1–SHE 8-15. 10.1029/2000JC000411.

[ref27] St Clair J. M.; Swanson A. K.; Bailey S. A.; Wolfe G. M.; Marrero J. E.; Iraci L. T.; Hagopian J. G.; Hanisco T. F. A New Non-Resonant Laser-Induced Fluorescence Instrument for the Airborne in Situ Measurement of Formaldehyde. Atmos. Meas. Tech. 2017, 10, 4833–4844. 10.5194/amt-10-4833-2017.

[ref28] St Clair J. M.; Swanson A. K.; Bailey S. A.; Hanisco T. F. CAFE: A New, Improved Nonresonant Laser-Induced Fluorescence Instrument for Airborne in Situ Measurement of Formaldehyde. Atmos. Meas. Tech. 2019, 12, 4581–4590. 10.5194/amt-12-4581-2019.

[ref29] Peck J.; Gonzalez L. A.; Williams L. R.; Xu W.; Croteau P. L.; Timko M. T.; Jayne J. T.; Worsnop D. R.; Miake-Lye R. C.; Smith K. A. Development of an Aerosol Mass Spectrometer Lens System for PM2.5. Aerosol Sci. Technol. 2016, 50, 781–789. 10.1080/02786826.2016.1190444.

[ref30] Hu W.; Campuzano-Jost P.; Day D. A.; Croteau P.; Canagaratna M. R.; Jayne J. T.; Worsnop D. R.; Jimenez J. L. Evaluation of the New Capture Vaporizer for Aerosol Mass Spectrometers (AMS) through Field Studies of Inorganic Species. Aerosol Sci. Technol. 2017, 51, 735–754. 10.1080/02786826.2017.1296104.

[ref31] Joo T.; Chen Y.; Xu W.; Croteau P.; Canagaratna M. R.; Gao D.; Guo H.; Saavedra G.; Kim S. S.; Sun Y.; Weber R.; Jayne J.; Ng N. L. Evaluation of a New Aerosol Chemical Speciation Monitor (ACSM) System at an Urban Site in Atlanta, GA: The Use of Capture Vaporizer and PM2.5 Inlet. ACS Earth Space Chem. 2021, 5, 2565–2576. 10.1021/acsearthspacechem.1c00173.

[ref32] Kakavas S.; Pandis S. N.; Nenes A. ISORROPIA-Lite: A Comprehensive Atmospheric Aerosol Thermodynamics Module for Earth System Models. Tellus B 2022, 74, 1–23. 10.16993/tellusb.33.

[ref33] Fountoukis C.; Nenes A. ISORROPIA II: A Computationally Efficient Thermodynamic Equilibrium Model for K+–Ca2+–Mg2+–NH+4 −Na+–SO24––NO–3 −Cl––H2O Aerosols. Atmos. Chem. Phys. 2007, 21.

[ref34] Petters M. D.; Kreidenweis S. M. A Single Parameter Representation of Hygroscopic Growth and Cloud Condensation Nucleus Activity. Atmos. Chem. Phys. 2007, 7, 1961–1971. 10.5194/acp-7-1961-2007.

[ref35] Cerully K. M.; Bougiatioti A.; Hite J. R.; Guo H.; Xu L.; Ng N. L.; Weber R.; Nenes A. On the Link between Hygroscopicity, Volatility, and Oxidation State of Ambient and Water-Soluble Aerosols in the Southeastern United States. Atmos. Chem. Phys. 2015, 15, 8679–8694. 10.5194/acp-15-8679-2015.

[ref36] Cerully K. M.; Raatikainen T.; Lance S.; Tkacik D.; Tiitta P.; Petäjä T.; Ehn M.; Kulmala M.; Worsnop D. R.; Laaksonen A.; Smith J. N.; Nenes A. Aerosol Hygroscopicity and CCN Activation Kinetics in a Boreal Forest Environment during the 2007 EUCAARI Campaign. Atmos. Chem. Phys. 2011, 11, 12369–12386. 10.5194/acp-11-12369-2011.

[ref37] Bougiatioti A.; Bezantakos S.; Stavroulas I.; Kalivitis N.; Kokkalis P.; Biskos G.; Mihalopoulos N.; Papayannis A.; Nenes A. Biomass-Burning Impact on CCN Number, Hygroscopicity and Cloud Formation during Summertime in the Eastern Mediterranean. Atmos. Chem. Phys. 2016, 16, 7389–7409. 10.5194/acp-16-7389-2016.

[ref38] Fountoukis C.; Nenes A.; Sullivan A.; Weber R.; Reken T. V.; Fischer M.; Matıas E.; Moya M.; Farmer D.; Cohen R. C. Thermodynamic Characterization of Mexico City Aerosol during MILAGRO 2006. Atmos. Chem. Phys. 2009, 9, 2141–2156. 10.5194/acp-9-2141-2009.

[ref39] Guo H.; Liu J.; Froyd K. D.; Roberts J. M.; Veres P. R.; Hayes P. L.; Jimenez J. L.; Nenes A.; Weber R. J. Fine Particle PH and Gas–Particle Phase Partitioning of Inorganic Species in Pasadena, California, during the 2010 CalNex Campaign. Atmos. Chem. Phys. 2017, 17, 5703–5719. 10.5194/acp-17-5703-2017.

[ref40] Guo H.; Nenes A.; Weber R. J. The Underappreciated Role of Nonvolatile Cations in Aerosol Ammonium-Sulfate Molar Ratios. Atmos. Chem. Phys. 2018, 18, 17307–17323. 10.5194/acp-18-17307-2018.

[ref41] Guo H.; Xu L.; Bougiatioti A.; Cerully K. M.; Capps S. L.; Hite J. R. J.; Carlton A. G.; Lee S.-H.; Bergin M. H.; Ng N. L.; Nenes A.; Weber R. J. Fine-Particle Water and PH in the Southeastern United States. Atmos. Chem. Phys. 2015, 15, 5211–5228. 10.5194/acp-15-5211-2015.

[ref42] Nenes A.; Pandis S. N.; Weber R. J.; Russell A. Aerosol PH and Liquid Water Content Determine When Particulate Matter Is Sensitive to Ammonia and Nitrate Availability. Atmos. Chem. Phys. 2020, 20, 3249–3258. 10.5194/acp-20-3249-2020.

[ref43] Barahona D.; Nenes A. Parameterization of Cirrus Cloud Formation in Large-Scale Models: Homogeneous Nucleation. J. Geophys. Res.: Atmos. 2008, 113 (D11), 1–15. 10.1029/2007JD009355.

[ref44] Wu C.; Yu J. Z. Evaluation of Linear Regression Techniques for Atmospheric Applications: The Importance of Appropriate Weighting. Atmos. Meas. Tech. 2018, 11, 1233–1250. 10.5194/amt-11-1233-2018.

[ref45] Tran H.; Molders N. Numerical Investigations on the Contribution of Point Source Emissions to the PM2.5 Concentrations in Fairbanks; Alaska | Elsevier Enhanced Reader, 2012.

[ref46] Liu T.; Abbatt J. P. D. Oxidation of Sulfur Dioxide by Nitrogen Dioxide Accelerated at the Interface of Deliquesced Aerosol Particles. Nat. Chem. 2021, 13, 1173–1177. 10.1038/s41557-021-00777-0. 34594012

[ref47] Joyce P. L.; von Glasow R.; Simpson W. R. The Fate of NO2 Emissions Due to Nocturnal Oxidation at High Latitudes: 1-D Simulations and Sensitivity Experiments. Atmos. Chem. Phys. 2014, 14, 7601–7616. 10.5194/acp-14-7601-2014.

[ref48] Zobrist B.; Marcolli C.; Pedernera D. A.; Koop T. Do Atmospheric Aerosols Form Glasses?. Atmos. Chem. Phys. 2008, 8, 5221–5244. 10.5194/acp-8-5221-2008.

[ref49] Virtanen A.; Joutsensaari J.; Koop T.; Kannosto J.; Yli-Pirilä P.; Leskinen J.; Mäkelä J. M.; Holopainen J. K.; Pöschl U.; Kulmala M.; Worsnop D. R.; Laaksonen A. An Amorphous Solid State of Biogenic Secondary Organic Aerosol Particles. Nature 2010, 467, 824–827. 10.1038/nature09455. 20944744

