# Supplementary material for: Source and Chemistry of Hydroxymethanesulfonate (HMS) in Fairbanks, Alaska
Source: Environ Sci Technol. 2022 May 11;56(12):7657–67. doi: 10.1021/acs.est.2c00410 (PMC9227704; doi:10.1021/acs.est.2c00410)
Supplement: Supplementary file 1 — es2c00410_si_001.pdf [file es2c00410_si_001.pdf]

Supplementary Information for

Source and Chemistry of Hydroxymethanesulfonate (HMS) in Fairbanks, Alaska

James Campbell[1], Michael Battaglia[2], Kayane Dingilian[2], Meeta Cesler-Maloney[1], Jason M. St. Clair[3,4], Thomas F. Hanisco[3], Ellis Robinson[5], Peter DeCarlo[5], William Simpson[1], Athanasios Nenes[2,6,7], Rodney J. Weber[2], Jingqiu Mao[1]

[1] Geophysical Institute and Department of Chemistry and Biochemistry, University of Alaska Fairbanks, Fairbanks, AK, USA

[2] School of Earth and Atmospheric Sciences, Georgia Institute of Technology, Atlanta, GA, 30332, USA

[3] Atmospheric Chemistry and Dynamics Laboratory, NASA Goddard Space Flight Center, Greenbelt, MD, 20771, USA

[4] Joint Center for Earth Systems Technology, University of Maryland Baltimore County, Baltimore, MD, 21228, USA

[5] Department of Environmental Health and Engineering, Johns Hopkins University, Baltimore, MD, 21218, USA

[6] Center for the Study of Air Quality and Climate Change, Institute of Chemical Engineering Sciences, Foundation for Research and Technology Hellas, Patras 26504, Greece

[7] Laboratory of Atmospheric Processes and their Impacts, School of Architecture, Civil and Environmental Engineering, École Polytechnique Fédérale de Lausanne, Lausanne 1015, Switzerland

Correspondence: Jingqiu Mao (jmao2@alaska.edu) and Rodney Weber (rweber@eas.gatech.edu)

Content:

Figure S1. Locations of field measurement sites.

Figure S2. Example Chromatogram.

Figure S3. Liquid concentrations of sulfate vs. concentrations of HMS for HMS IC standards.

Figure S4. Calculation of sulfite and bisulfite concentrations.

Figure S5. Temperature comparison between NCore site and Fairbanks International Airport.

Figure S6. Diurnal cycle of ozone in January and February.

Figure S7. $PM_{2.5}$ ALWC vs. OA and ALWC vs. sulfate.

Figure S8. Calculated HMS production rate as a function of pH and ambient temperature.

Figure S9. Temperature frequency for the HMS measurement period.

Figure S10. Correlation between HMS and related measurements.

Figure S11. Comparison of $PM_{2.5}$ and HMS with NO_y

Figure S12. Calculated pH vs sulfate and ALWC vs. temperature.

Figure S13. Calculated pH vs. HMS concentrations.

Figure S14. Comparison between PILS and ACSM measurements.

Table S1. Moch et al. (2018) GEOS-Chem implementation.

Table S2. Average concentrations of NVCs during both sampling periods.

Number of pages: 18

Number of Figures: 14

Number of Tables: 2

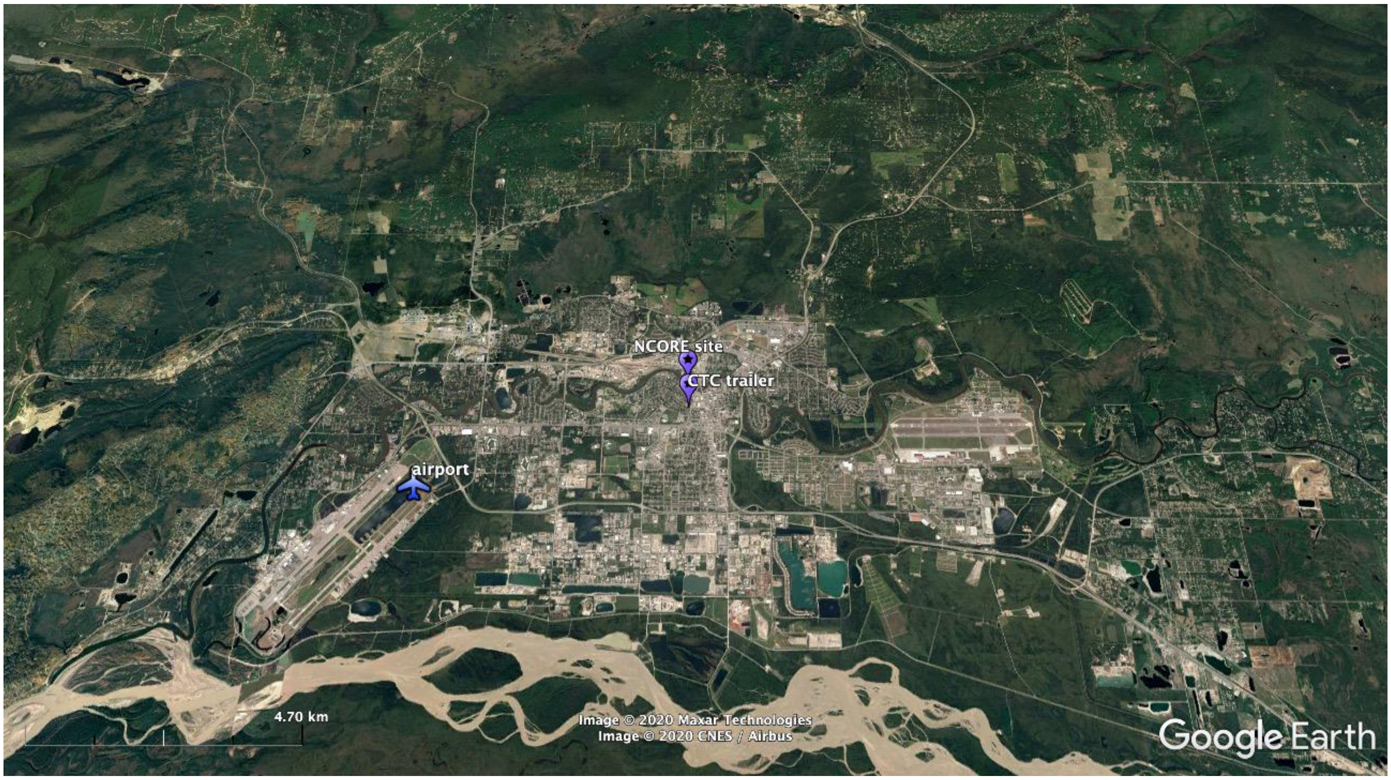

Figure S1. Locations of field measurement sites (same for both sampling periods). The NCore site is about 500 m away from CTC trailer, and Fairbanks International Airport is about 5 km away from CTC trailer.

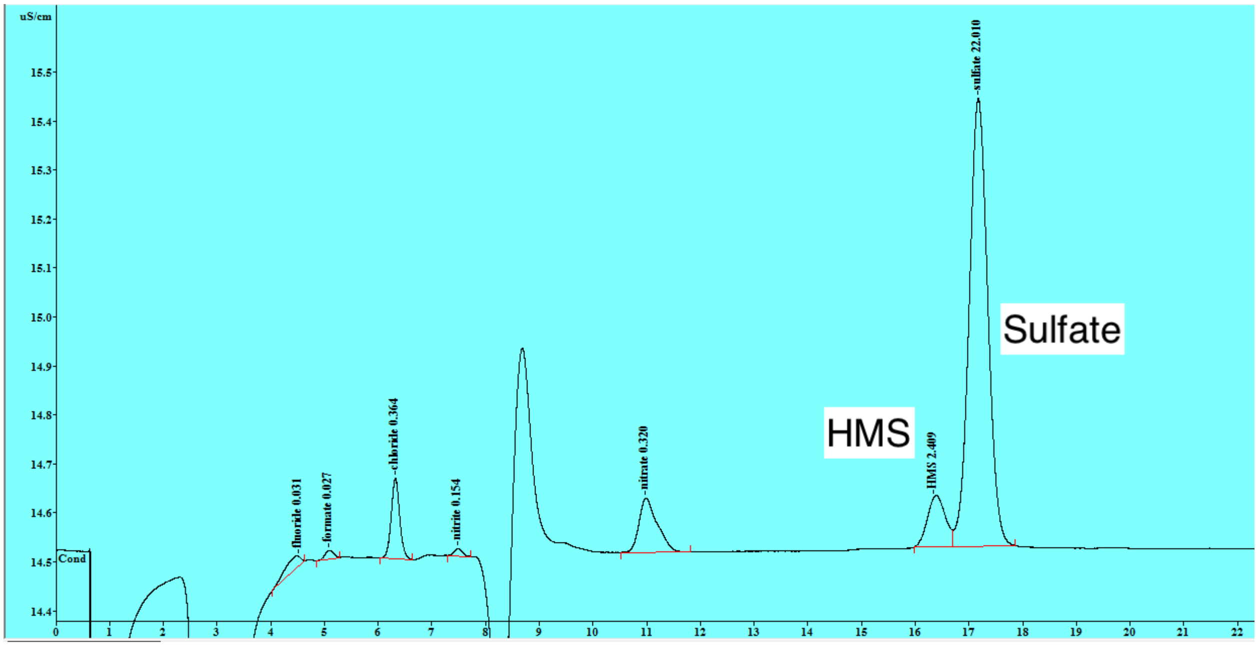

Figure S2. Example chromatogram. HMS and sulfate are shown to overlap on the right.

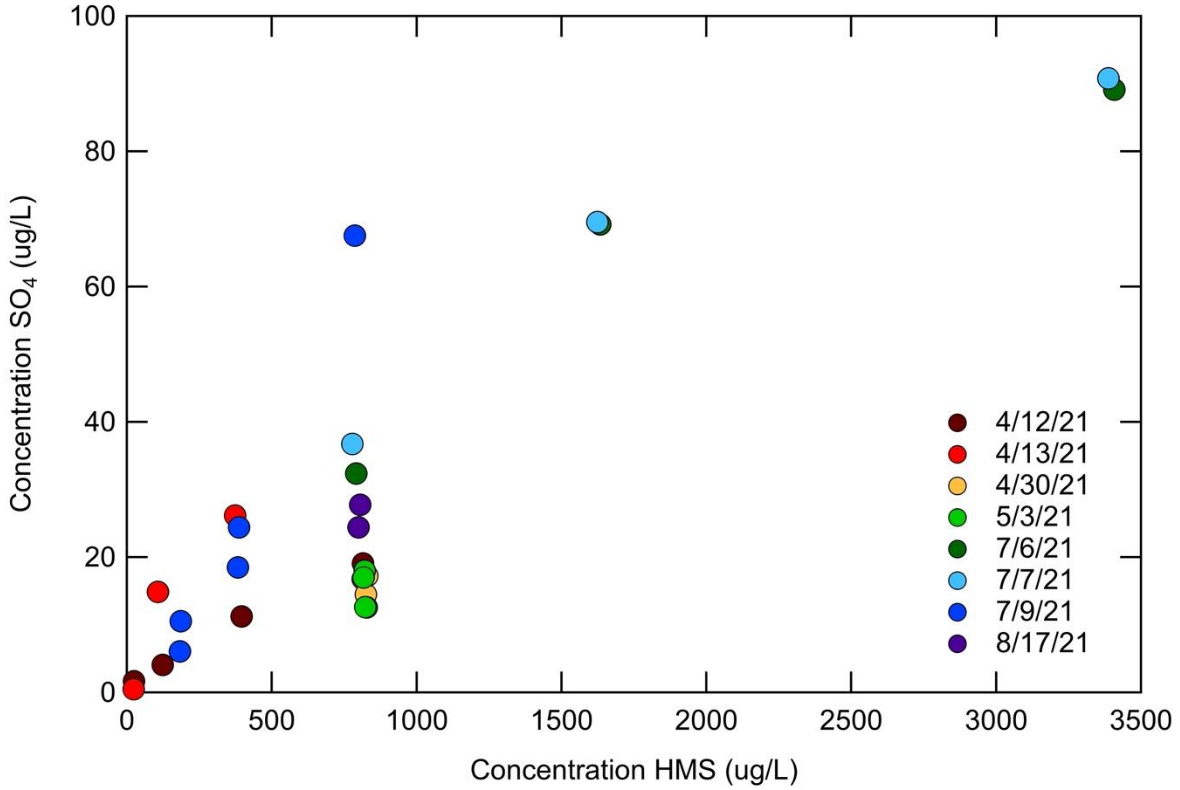

Figure S3. Liquid concentrations of sulfate vs. concentrations of HMS for HMS IC standards ranging from approximately 5 to 3500 µg/L.

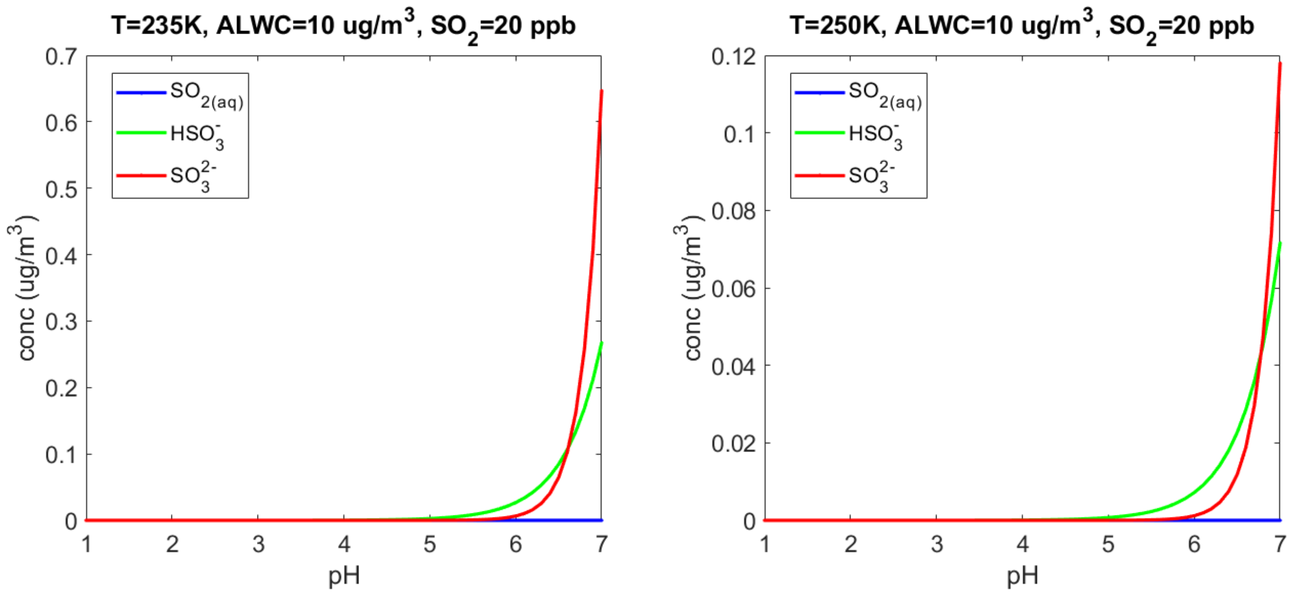

Figure S4. Thermodynamic calculation of aqueous SO_2, sulfite, and bisulfite concentrations over pH 1 to 7. Calculations were done for 235 K (left) and 250 K (right). ALWC = 10 μg/m^3, SO_2 = 20 ppb.

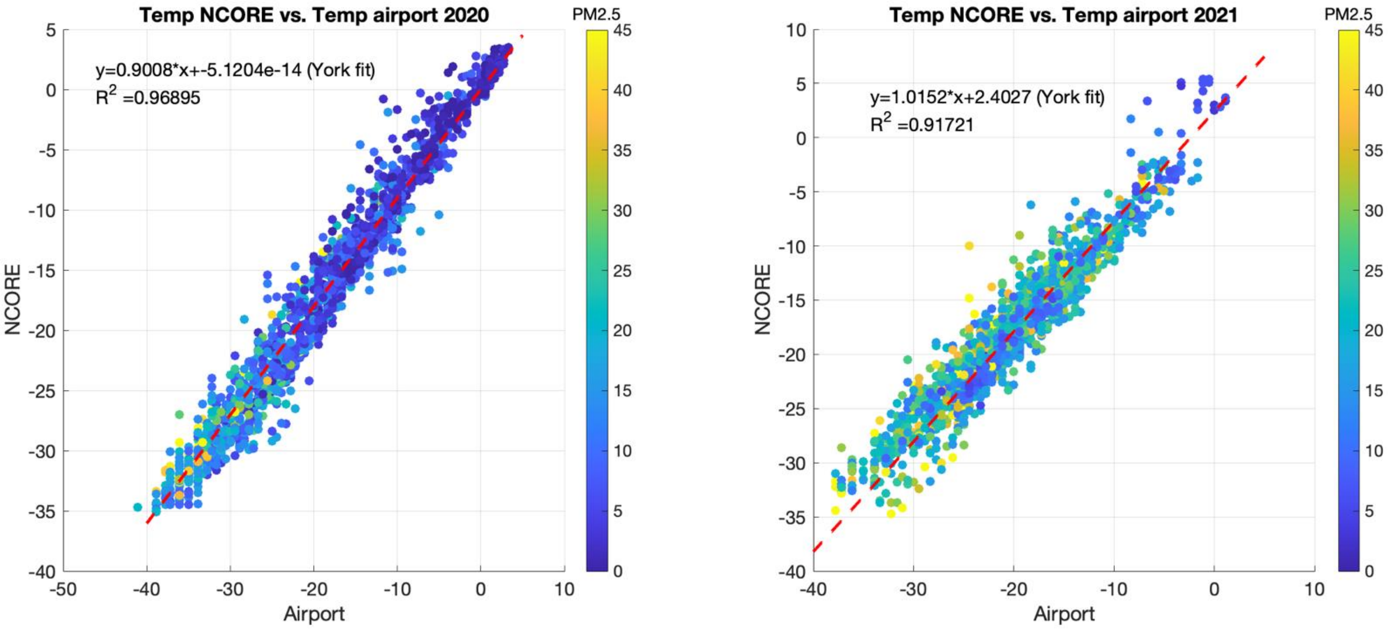

Figure S5. Temperature comparison between NCore site and Fairbanks International Airport.

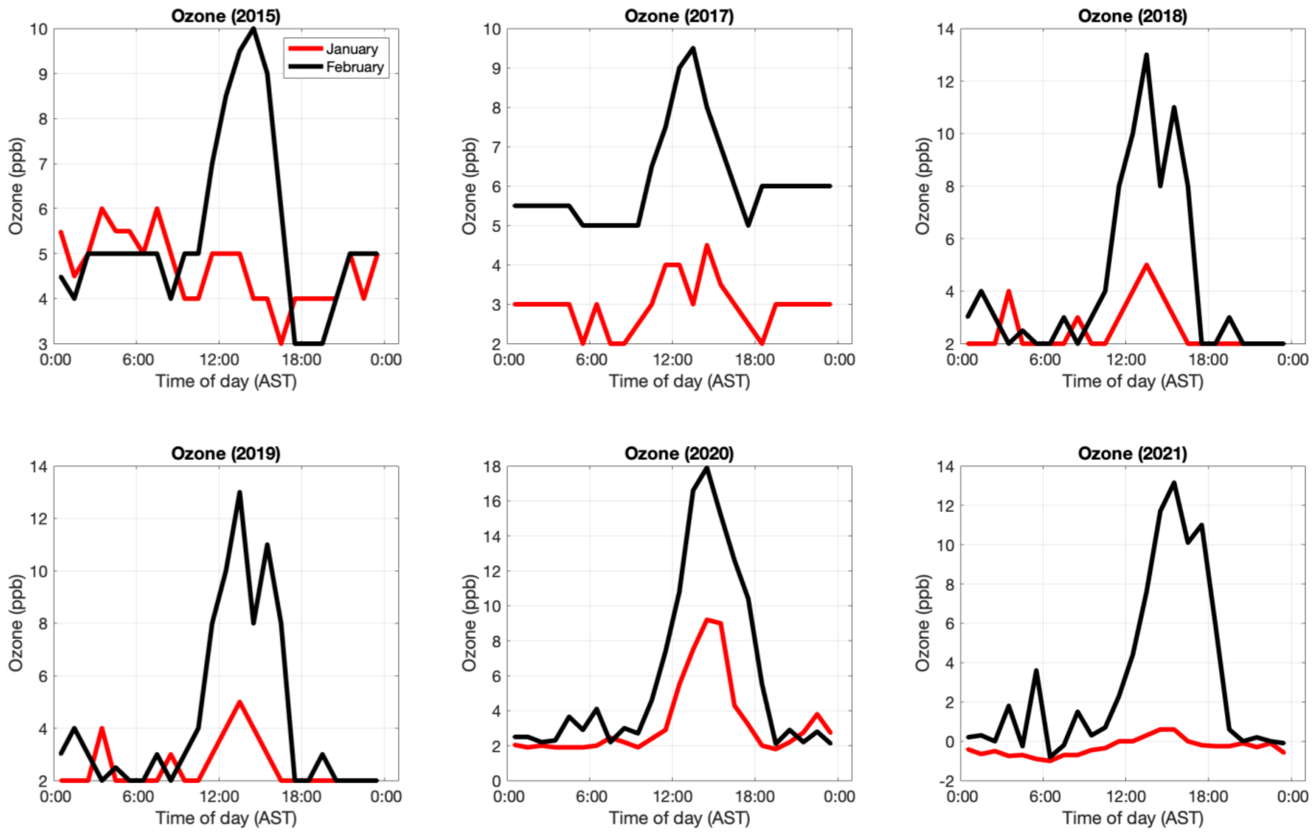

Figure S6. Diurnal cycle of ozone in January (red) and February (black) in different years.

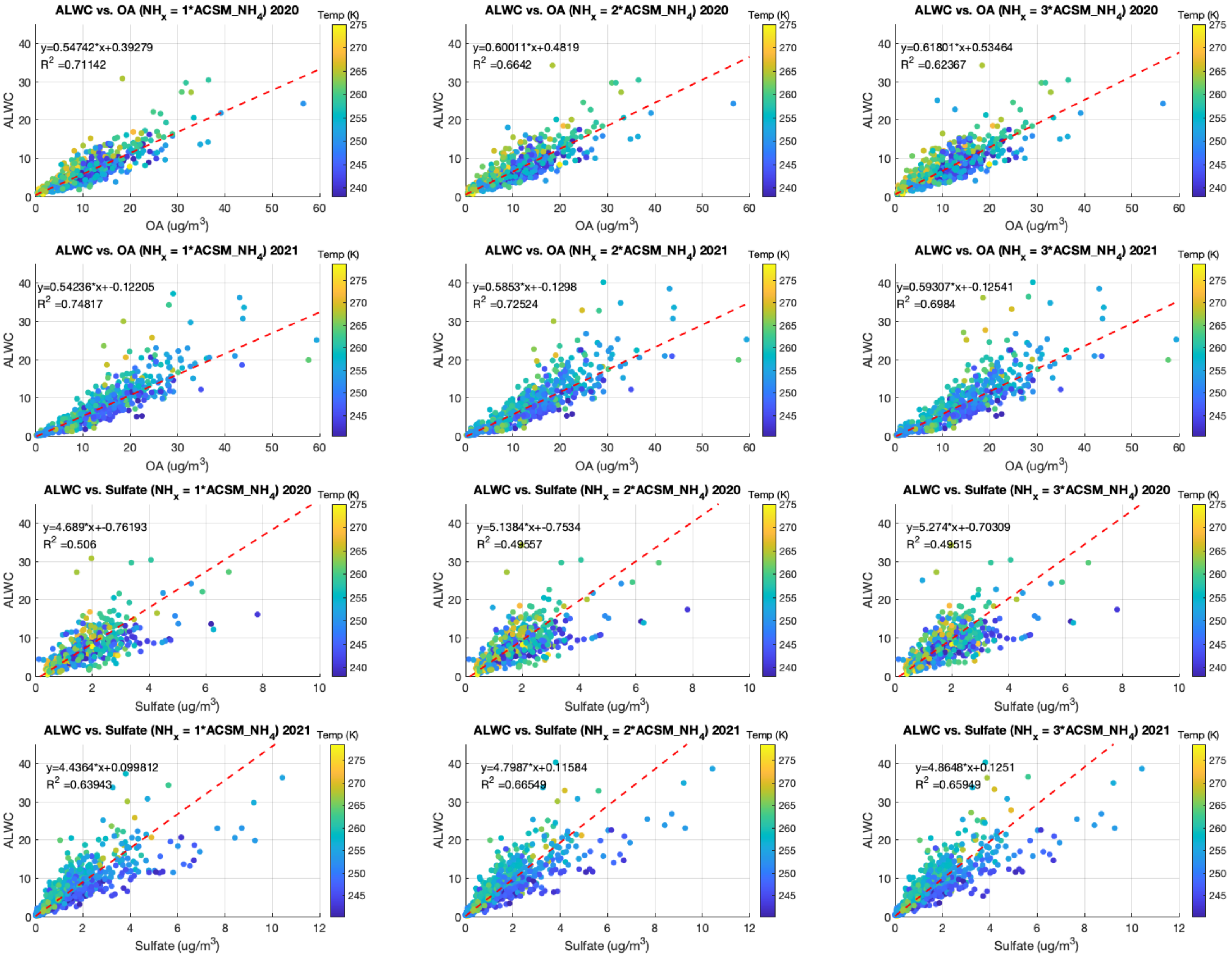

Figure S7. $PM_{2.5}$ ALWC vs OA and ALWC vs sulfate for κ = 0.15 for three different NH_x considerations in 2020 and 2021. Typical Fairbanks winter conditions are OA~12 μg/m^3, sulfate~2 μg/m^3, nitrate~0.7 μg/m^3, ammonium~0.4 μg/m^3, chloride~0.3 μg/m^3, RH~70-80%. Data points are colored by temperature.

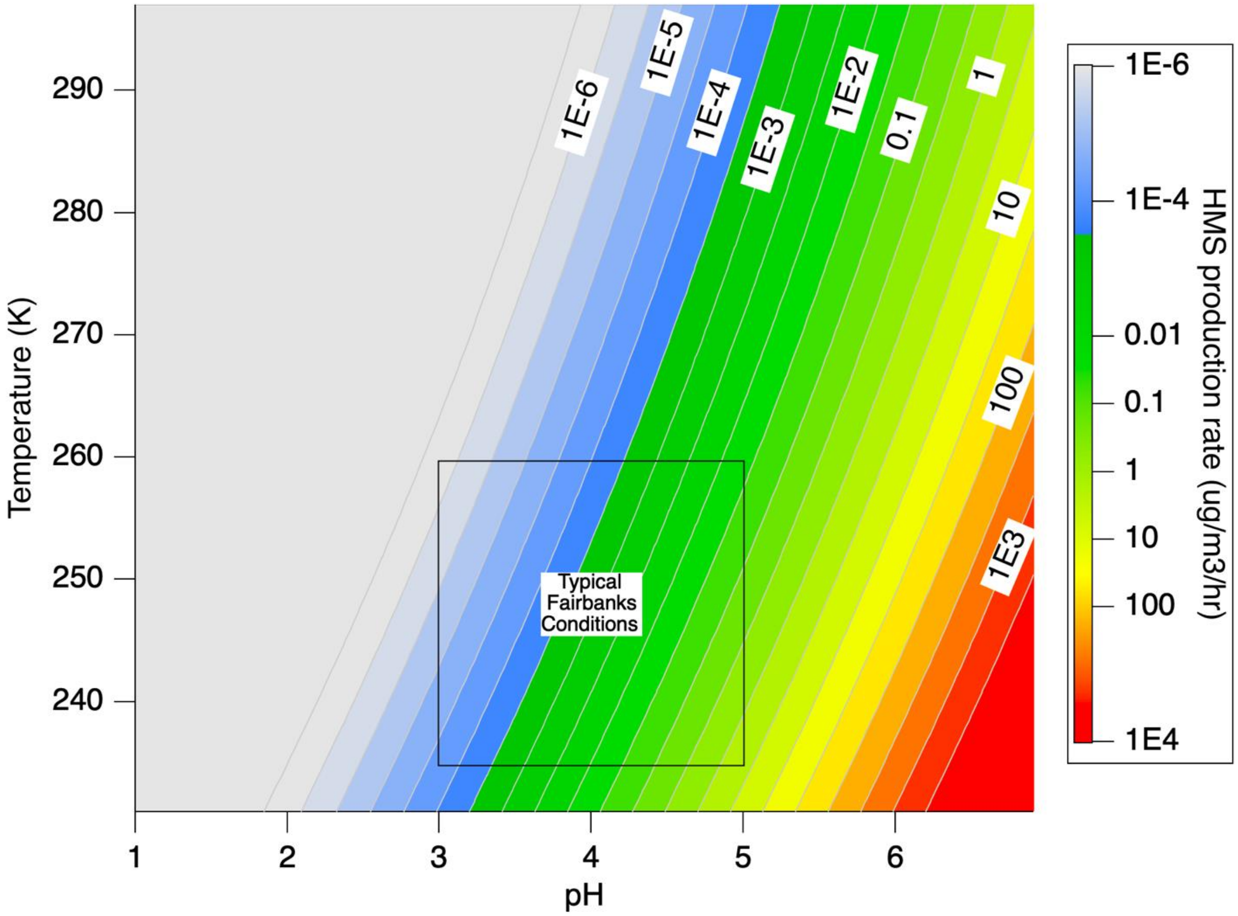

Figure S8. Calculated HMS production rate as a function of pH and ambient temperature. We assume SO_2 = 20 ppbv, HCHO = 5 ppbv, ALWC = 10 µg/m^3.

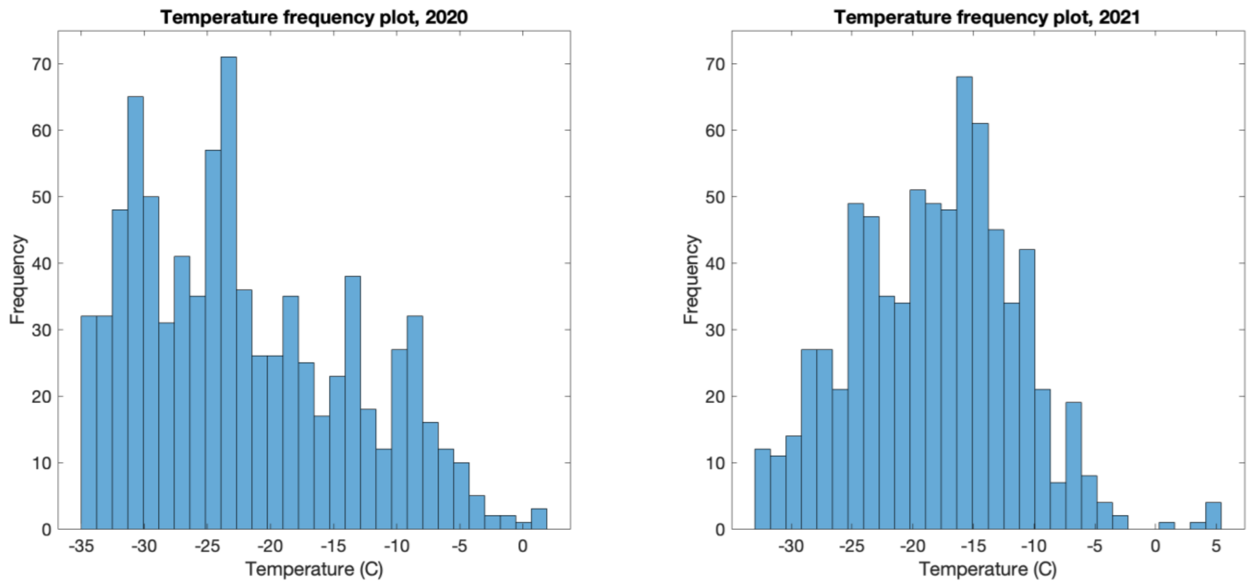

Figure S9. Temperature frequency for the HMS measurement period in both 2020 and 2021.

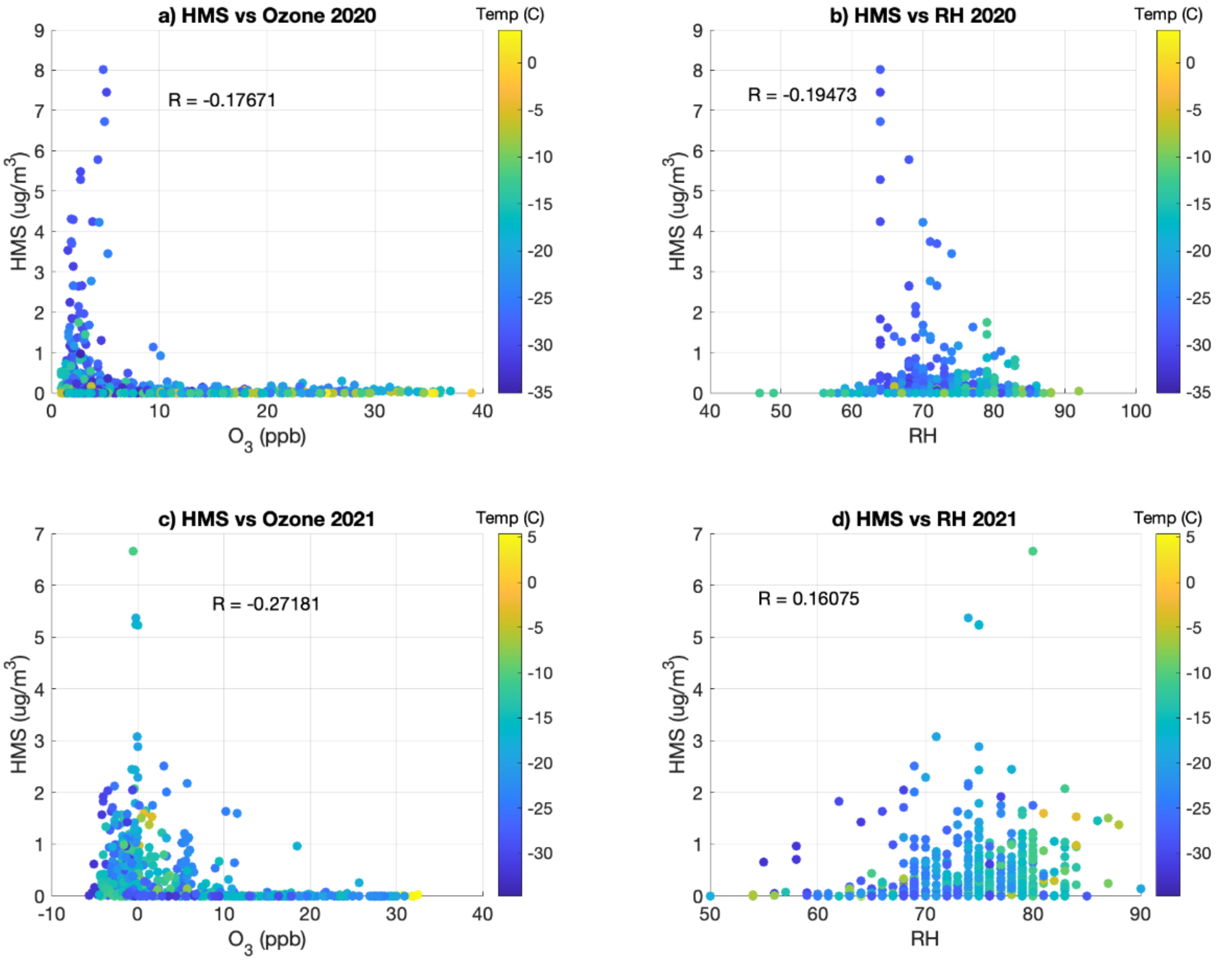

Figure S10. Correlation between HMS and related measurements.

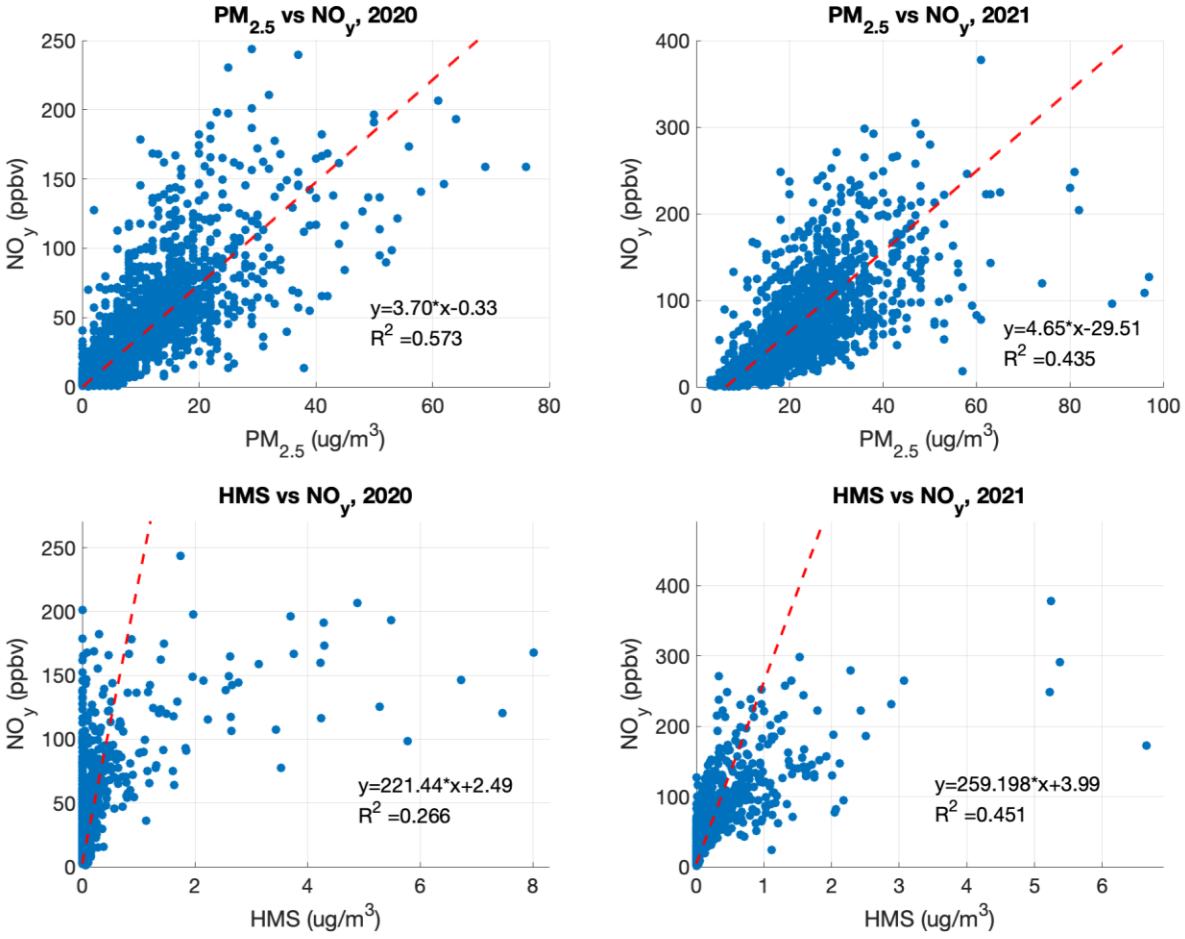

82 **Figure S11.** Comparison of $PM_{2.5}$ vs. NO_y (top) and HMS vs. NO_y (bottom).

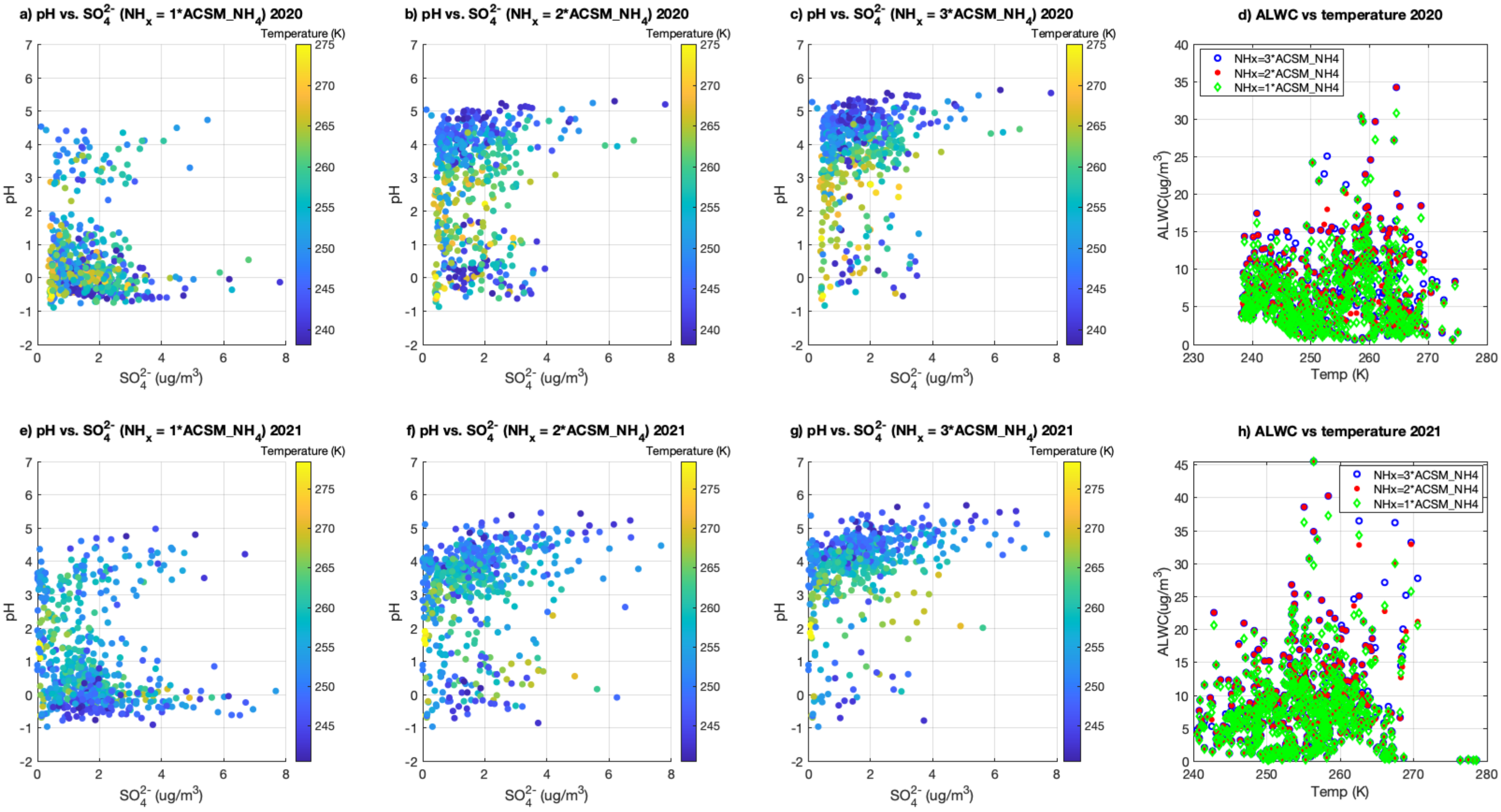

Figure S12. (a-c) and (e-g) show the dependence of pH on sulfate at different levels of NH_x in 2020 and 2021, colored by temperature. d and h show ALWC vs. temperature at different NH_x levels. All figures are for $\kappa = 0.15$. Typical Fairbanks winter conditions are OA~12 μg/m^3, sulfate~2 μg/m^3, nitrate~0.7 μg/m^3, ammonium~0.4 μg/m^3, chloride~0.3 μg/m^3, RH~70-80%.

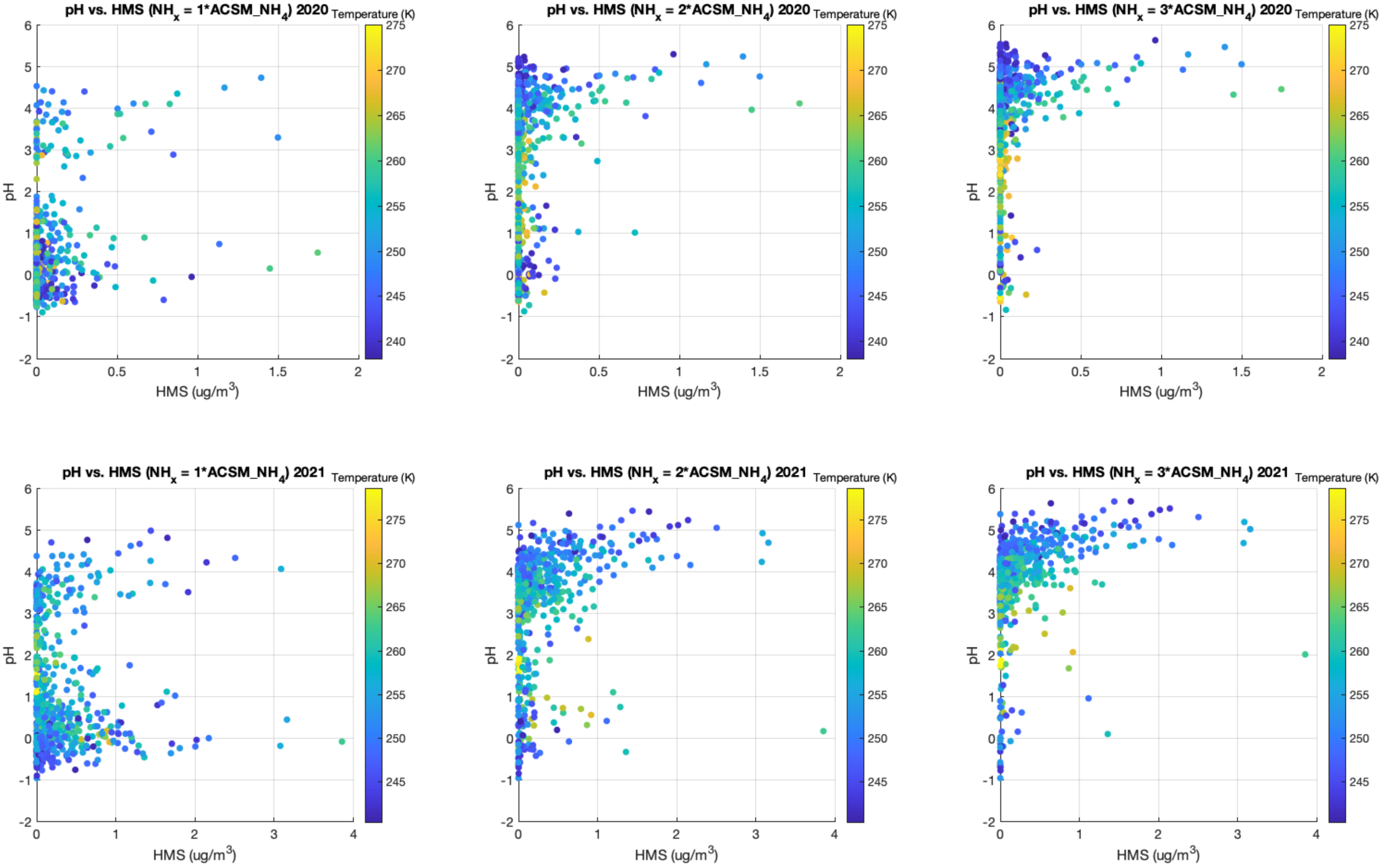

Figure S13. Calculated pH vs. HMS concentrations at κ = 0.15 and different levels of NH_x, colored by temperature. Typical Fairbanks winter conditions are OA~12 μg/m^3, sulfate~2 μg/m^3, nitrate~0.7 μg/m^3, ammonium~0.4 μg/m^3, chloride~0.3 μg/m^3, RH~70-80%.

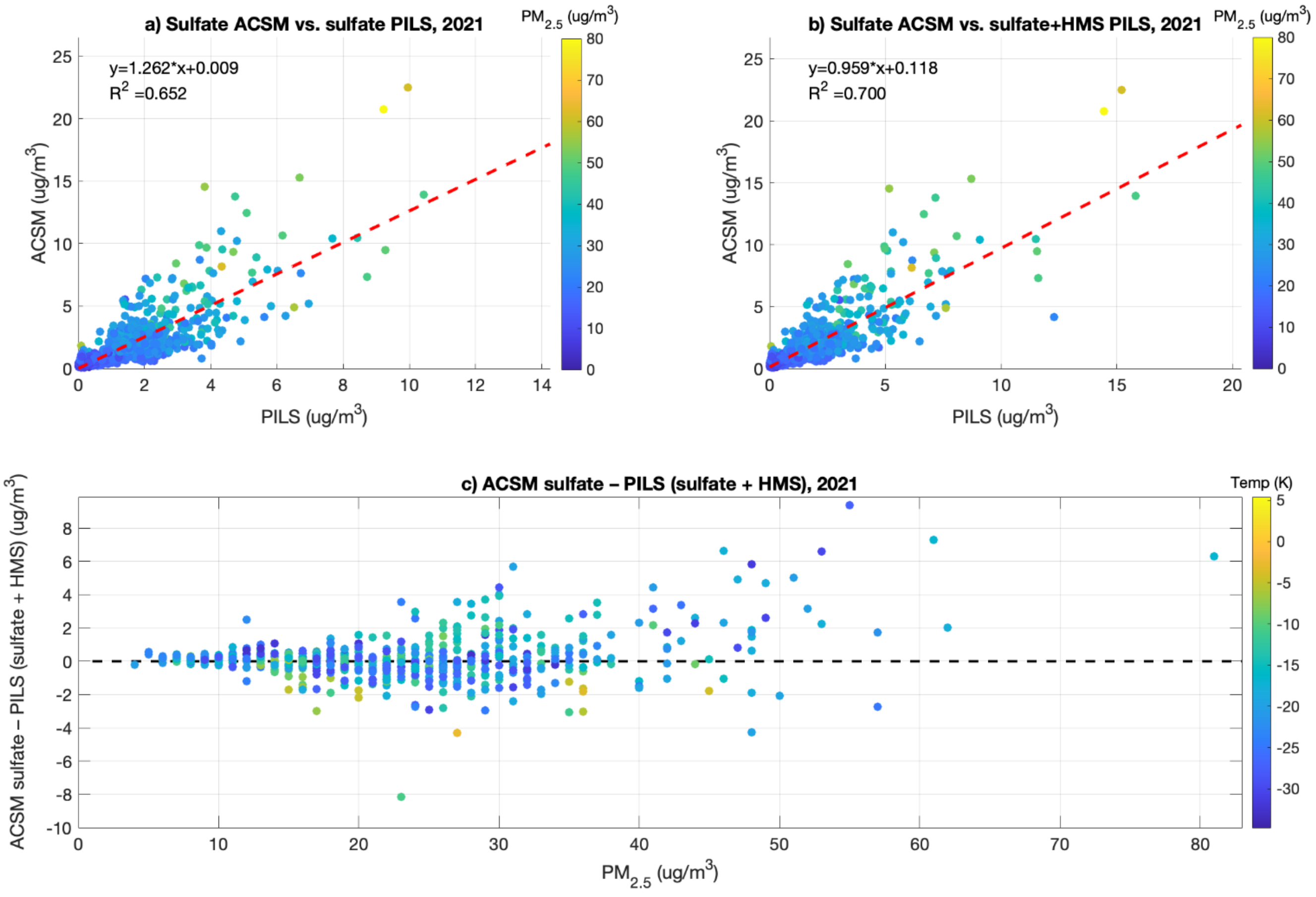

Figure S14. (a) ACSM sulfate vs. PILS sulfate. (b) ACSM sulfate vs. PILS sulfate + HMS. (c) ACSM sulfate – PILS (sulfate + HMS) vs $PM_{2.5}$. The discrepancy between the PILS and ACSM increases when $PM_{2.5}$ increases, even when HMS is included.

Table S1. *Moch et al. (2018) GEOS-Chem implementation. K_{298} is the rate constant, H_{298} is the Henry's law constant, and K_{eq} is the equilibrium constant, all at 298 K. -ΔH/R is the enthalpy of dissolution divided by the universal gas constant.*

Reaction	Variable	Value (units)	Reference
$HSO_3^- + HCHO_{(aq)}$ => HMS	K_{298}, -ΔH/R	7.9E2 (M^{-1} s^{-1}), -4900 (K)	Boyce and Hoffman, 1984; Seinfeld and Pandis, 2006
$SO_3^{2-} + HCHO_{(aq)}$ => HMS + OH^-	K_{298}, -ΔH/R	2.5E7 (M^{-1} s^{-1}), -1800 (K)	Boyce and Hoffman, 1984; Seinfeld and Pandis, 2006
$SO_{2(g)} + H_2O = SO_2{\cdot}H_2O$	H_{298}, -ΔH/R	1.23 (M atm^{-1}), 3145 (K)	Seinfeld and Pandis, 2006; Jacob 1986
$SO_2{\cdot}H_2O = HSO_3^- + H^+$	K_{eq}, -ΔH/R	1.3E-2 (M), 2000 (K)	Munger et al., 1983; Seinfeld and Pandis, 2016
$HSO_3^- = SO_3^{2-} + H^+$	K_{eq}, -ΔH/R	6.31E-8 (M), 1500 (K)	Seinfeld and Pandis, 2016
$HCHO_{(g)} = HCHO_{(aq)}$	H_{298}, -ΔH/R	2.5 (M atm^{-1}), 6800 (K)	Seinfeld and Pandis, 2016
$HCHO_{(aq)} = HCH(OH)_2$	K_{eq}, -ΔH/R	2530 (M), 4020 (K)	Seinfeld and Pandis, 2016

Table S2. *Average concentrations of calcium, magnesium, potassium, and sodium during both sampling periods in µg/m³. Potassium ion and sodium ion were measured using IC, and everything else was measured with X-ray fluorescence (XRF).*

Year	Calcium	Magnesium	Potassium	Potassium ion	Sodium	Sodium ion
2020	0.032	0.024	0.101	0.073	0.093	0.056
2021	0.039	0.002	0.336	0.307	0.091	0.033
